# Cyclic Octapeptides Composed of Two Glutathione Units Outperform the Monomer in Lead Detoxification

**DOI:** 10.1002/cmdc.202200152

**Published:** 2022-05-24

**Authors:** Luca Sauser, Tadeáš Kalvoda, Ayça Kavas, Lubomír Rulíšek, Michal S. Shoshan

**Affiliations:** ^1^ Department of Chemistry University of Zurich Winterthurerstrasse 190 8057 Zurich Switzerland; ^2^ Institute of Organic Chemistry and Biochemistry Czech Academy of Sciences Flemingovo náměstí 2 16610 Praha 6 Czech Republic

**Keywords:** Lead, Peptides, Chelates, Glutathione, Bioinorganic chemistry

## Abstract

A rationally‐designed scaffold of cyclic octapeptides composed of two units of the natural tripeptide glutathione (GSH) was optimized to strongly and selectively capture toxic lead ions (Pb(II)). Using state‐of‐the‐art computational tools, a list of eleven plausible peptides was shortened to five analogs based on their calculated affinity to Pb(II) ions. We then synthesized and investigated them for their abilities to recover Pb‐poisoned human cells. A clear pattern was observed from the *in vitro* detoxification results, indicating the importance of cavity size and polar moieties to enhance metal capturing. These, together with the apparent benefit of cyclizing the peptides, improved the detoxification of the two lead peptides by approximately two folds compared to GSH and the benchmark chelating agents against Pb poisoning. Moreover, the two peptides did not show any toxicity and, therefore, were thoroughly investigated to determine their potential as next‐generation remedies for Pb poisoning.

## Graphical abstract

**Glutathione was incorporated** into rationally‐designed cyclic octapeptides to enhance its binding affinity towards lead (Pb) and detoxify it. We computationally screened different variations of the scaffold and identified promising binders, five of which were subjected to intensive investigation. This included *in vitro* testing, which revealed their enhanced Pb‐detoxification capability due to high Pb‐affinity and selectivity, together with lack of toxicity.

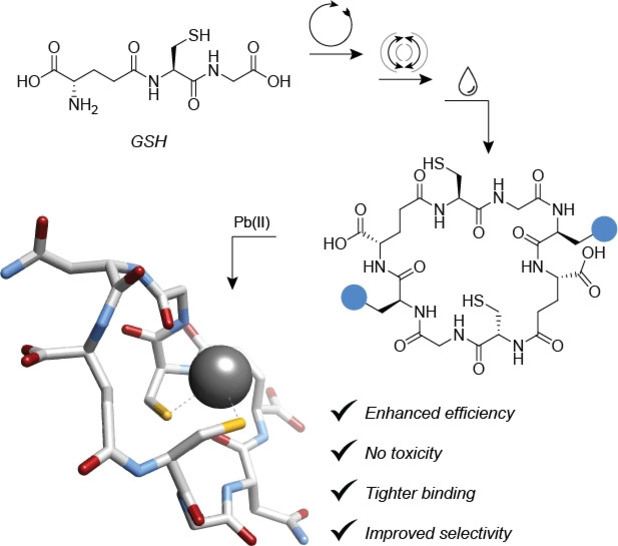

## Introduction

Lead (Pb) is a non‐essential, toxic metal widely distributed in the environment. More than one million deaths are attributed to Pb poisoning annually.^[1]^ Pb exposure most often results from ingestion of contaminated foods, beverages, or dust,^[1]^ as well as inhalation of polluted air.^[2]^ Upon absorption, Pb enters the bloodstream, from where it is distributed to the soft tissues, such as the liver, kidneys, and the brain, and subsequently deposited in the mineralized tissues.[^2^, ^3^] In adults, more than 95 % of Pb is eventually stored in bones and teeth,^[3]^ with a biological half‐life of 20–30 years and can be released back into the bloodstream.^[4]^

Pb(II) ion, which is the most common Pb oxidation state,^[5]^ exerts toxicity by binding to proteins, changing their structure, and rendering them dysfunctional.^[6]^ Among the most prevalent coordinating groups in proteins are thiols and carboxylic acids.[^6^, ^7^, ^8^] Pb(II) ion can also outcompete essential metal ions for binding sites, altering metalloenzyme activity or impairing metal ion transport.[^6^, ^9^] In addition, exposure to Pb elevates oxidative stress by covalent binding to and subsequent inactivation of antioxidants and antioxidant enzymes, such as glutathione (GSH) and superoxide dismutase (SOD).^[10]^ Effects of Pb poisoning primarily manifest in the renal, the haematopoietic, and the nervous systems, with the latter being the most vulnerable target.[^6^, ^9^] In the brain, effects also result from Pb interfering with Ca‐dependent processes, such as neurotransmission.[^4^, ^11^] As Pb(II) is capable of disrupting neurological development, the nervous system of children is particularly prone to sustaining long‐term damage,[^1^, ^12^] and exposure to Pb during the prenatal period or childhood has been associated with persisting cognitive deficits and behavioral problems.[^3^, ^4^, ^12^]

The primary medical treatment for reducing the toxic effects of Pb is chelation therapy, where small organic molecules are applied to convert biologically bound Pb into a complex called a chelate.[^6^, ^13^, ^14^] An ideal chelating agent (CA) should fulfill several criteria: selectivity for the respective metal ion, solubility in the physiological environment of both the *apo* and the *holo* compounds, low toxicity, ability to penetrate cellular membranes, formation of a non‐toxic complex, and effective clearance of the complex from the body.[^9^, ^13^] Frequently used CAs for detoxifying Pb are *ethylenediaminetetraacetic acid* (EDTA) and *dimercapto‐succinic acid* (DMSA), which can reduce the total burden of Pb in the body.[^12^, ^15^] Despite their relative efficacy, these drugs present clinically significant obstacles. Most notably, the chelation and depletion of essential metal ions during treatment have been reported as issues of chelation therapy.[^9^, ^12^, ^14^] Further, not all body compartments are accessible to CAs. EDTA and DMSA have an extracellular distribution, rendering them unable to chelate intracellular Pb.[^9^, ^14^] Moreover, EDTA was noted to redistribute Pb to the brain, which is more sensitive than other organs to Pb exposure.[^9^, ^14^] Considering all these disadvantages, the standard CAs for Pb poisoning are only indicated for extremely high blood‐lead levels (BLL) of 9–14 times the BLL determined to require intervention,[^14^, ^16^] even though there is no known safe BLL.[^1^, ^3^]

Inspired by natural detoxification systems, and based on our recent accomplishments with cyclic tetrapeptides^[17]^ and GSH‐analogs,^[18]^ we envisioned that two units of GSH cyclized together would provide the necessary macrocyclic cavity and enough binding moieties to strongly capture Pb(II) ions. The cyclic design was chosen to improve metabolic stability^[19]^ and for imposing conformational constraints on the peptide to achieve a more defined three‐dimensional structure.^[20]^

In this study, eleven peptide analogs were investigated computationally and ranked based on their complexation stability with Pb(II) ions. We then synthesized the lead members and tested them for their ability to recover Pb‐poisoned human cells. A thorough investigation of the two best candidates reveals the potential of our original scaffold design and the correlation between computed complexation free energy and the recovery of cells poisoned by Pb(II) ions.

## Results and Discussion

### Computationally aided scaffold design

**Figure 1:**
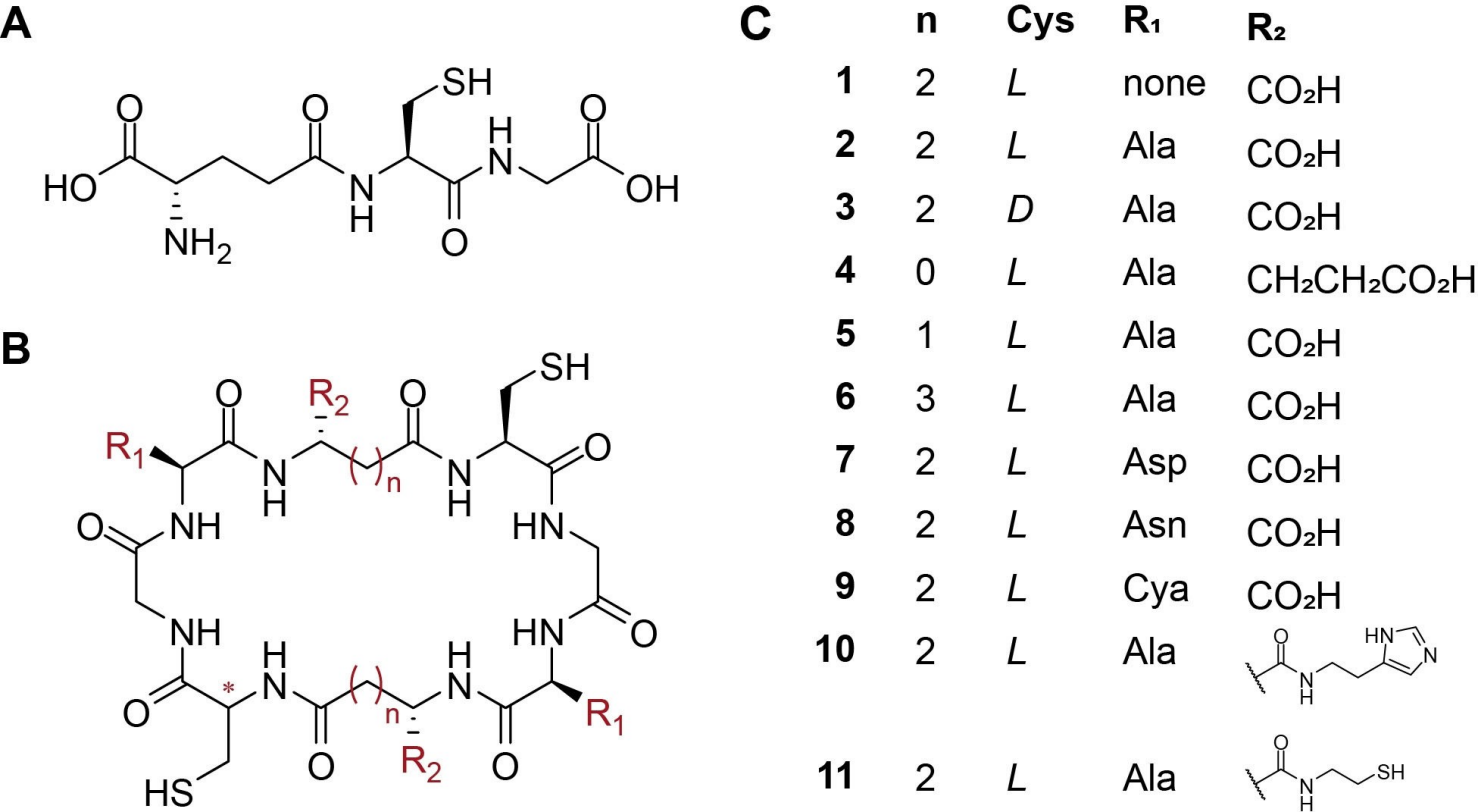
(A) Glutathione (GSH), (B) cyclic peptide scaffold investigated in this work, and (C) the eleven peptides that were subjected to computational investigation on their affinity to Pb(II) ions.

**Figure 2:**
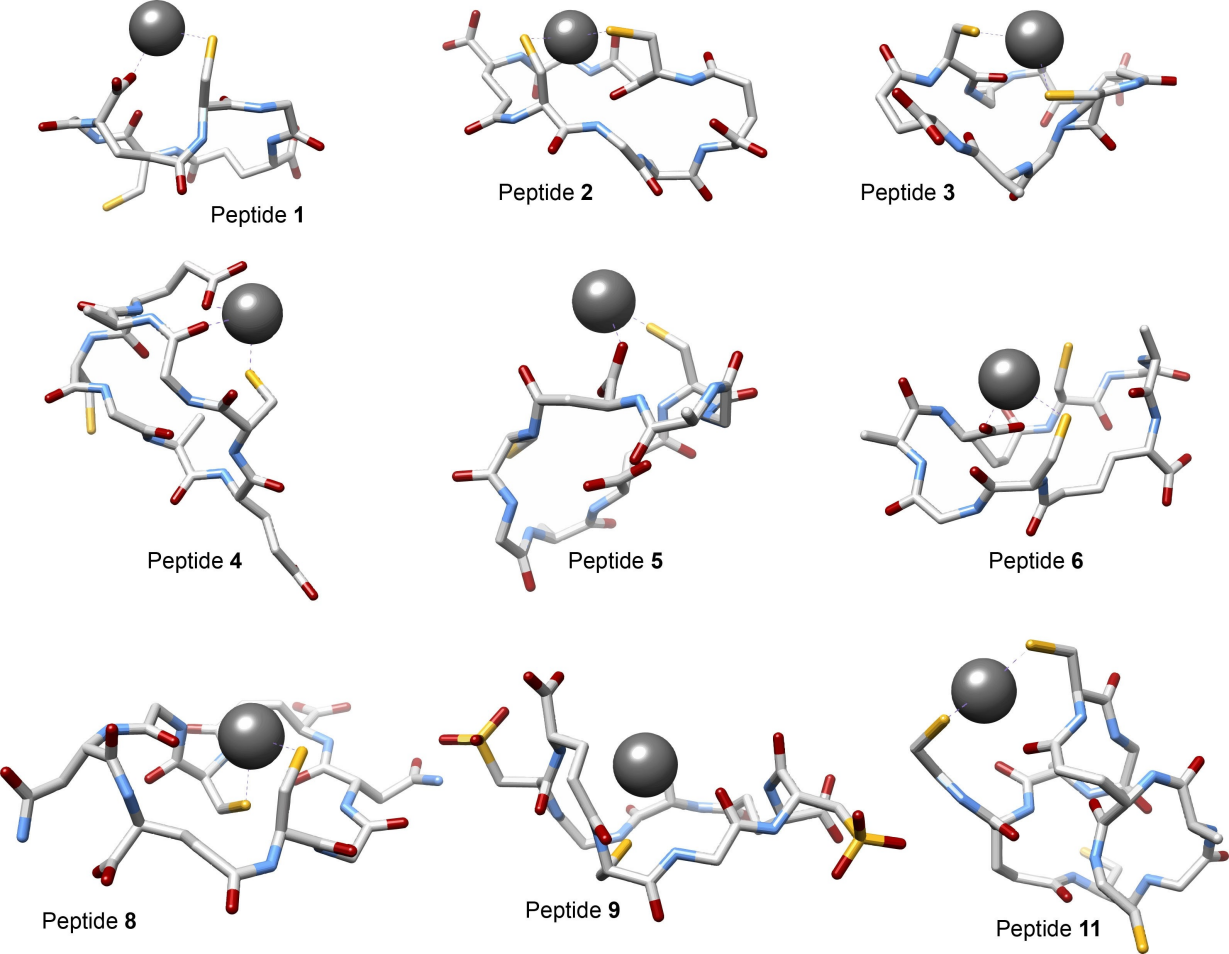
Calculated lowest‐energy structures of [Pb‐**X**⋅(H_2_O)_m_]⋅n{H_2_O} where X is nine selected peptides among the investigated eleven and m+n=4. Hydrogen atoms and four water molecules, placed in the first or the second coordination spheres, were omitted for clarity.

We aimed to investigate whether a head‐to‐tail‐cyclized peptide scaffold containing two units of GSH would increase the low affinity of GSH to Pb(II) ions (peptide **1**). We, therefore, used QM(DFT‐D3)//COSMO‐RS calculations employed and calibrated in our previous work[^17^, ^18^, ^21^] to determine the most stable equilibrium structure of the [Pb‐**1**⋅(H_2_O)_*m*_]⋅*n*{H_2_O} complex (Figures 1–2; Table 1). **1** was predicted to be a very poor binder of Pb(II), as reflected in its computed free energy of complexation, ΔG_*comp*_.^[22]^ To improve the scaffold and shed light on the reasons that make **1** a poor ligand for Pb(II) ions, we repeated the calculation with an octapeptide analog that contains two additional alanine (Ala) residues spacing the two GSH units (peptide **2**). Ala was chosen as the simplest yet chiral proteinogenic amino acid. The extension of the macrocycle was proved effective as the ΔG_*comp*_ was shifted to a more favorable value, which is comparable to GSH and yet offers more potential for tuning the scaffold.

**Table 1:** Peptides investigated herein and their computed complexation Gibbs free energy values that are the Δ*G* for the reaction: [Pb(H_2_O)_*5*_]+**X**→[Pb‐**X**⋅(H_2_O)_*m*_]⋅*n*{H_2_O}+(5‐*m*‐*n*)H_2_O.

	Change^[a]^	Motivation	Δ*G_comp_* [kcal mol^−1^]
**GSH**	‐	–	−1.2^[18]^
**GSH**	2 : 1 GSH:Pb(II)	–	23.2
**1**	–	“WT”	13.9
**2**	Octamer *vs*. hexamer	Larger cavity and binding	−1.0
**3**	*L*Cys6→*D*Cys6	Cys stereochemistry	6.2
**4**	γGlu→αGlu	Macrocycle cavity size	10.5
**5**	γGlu→βAsp		−6.4
**6**	γGlu→δhGlu		−2.3
**7**	Ala→Asp	Polarity	3.6
**8**	Ala→Asn		−6.1
**9**	Ala→Cya^[b]^		−8.8
**10**	CO_2_H_(γGlu)_→CONH_(histamine)_	Further binding	33.0
**11**	CO_2_H_(γGlu)_→CONH_(cysteamine)_		42.0

[a] Unless differently stated, the change occurred in both residues. [b] Cysteic acid.

We then varied four structural and functional parameters of this basic octameric scaffold to further optimize Pb(II) binding *in silico*, which consisted of (a) inverting cysteine (Cys) stereochemistry; (b) varying the macrocycle size by contracting or expanding the backbone carbon skeleton of γ‐glutamic acid (γGlu); (c) replacing Ala with other amino acids (AAs) to tune properties, such as polarity and coordinating moieties; and (d) modifying functionalization of the scaffold to increase binding sites by coupling additional entities to γGlu free carboxylic acid.

As expected, in the most stable isomer of the [Pb‐**2**⋅(H_2_O)_*m*_]⋅*n*{H_2_O} complex (predicted computationally), both Cys participate in binding the ion (Figure 2). Likewise, the thiolato group of GSH also coordinates with Pb(II) ion.[^18^, ^23^, ^24^] We, therefore, inverted the stereochemistry of one of the Cys residues of **2** to form peptide **3**, aiming to further understand the effect of Cys configuration on Pb‐binding and stability. Computing the equilibrium structure of the [Pb‐**3**⋅(H_2_O)_*m*_]⋅*n*{H_2_O} complex (Figure 2) revealed no dramatic impact on Pb‐coordination mode. Yet, the increase in ΔG_*comp*_ by 7.2 kcal mol^−1^ (less favorable binding) upon inversion of one Cys to its *D* isomer indicates that the preorganization of the Cys residues in the all‐*L* scaffold is more favorable and was therefore adopted as the default configuration for subsequent structures.

We then screened various macrocyclic sizes in which the backbone was contracted or expanded by replacing γGlu with α‐glutamic acid (αGlu; peptide **4**), β‐aspartic acid (βAsp; peptide **5**), or δ‐homoglutamic acid (δhGlu; peptide **6**; Figure 1). Both expansion and contraction by one methylene group resulted in a decrease in the computed ΔG_*comp*_, with contraction offering an approximately threefold reduction compared to expansion (Figure 2; Table 1). Interestingly, the most stable complexation modes with **5** and **6** were revealed to be through coordination of one thiolate and the carboxylate of the opposite GSH subunit (Figure 2). A similar binding mode was also found in the case of **4** but resulted in a dramatic destabilization upon complexation (Figure 2; Table 1). The increase in side‐chain flexibility of **4** did not compensate for the decrease in degrees of freedom of the macrocycle. This, in addition to the steric interruption resulted from the side‐chains destabilizes the complex.

Since cyclization of the peptides neutralizes the charges at the N‐ and C‐termini, the aqueous solubility should be optimized, as it is a crucial parameter for achieving therapeutic efficacy.^[25]^ We, therefore, studied the effect of replacing Ala with more polar AAs: aspartic acid (Asp; peptide **7**), asparagine (Asn; peptide **8**), and the noncanonical AA (ncAA) cysteic acid (Cya; peptide **9**), that harbors a sulfonate group on its side‐chain, which aside of its high polarity can serve as an additional binder of Pb(II) ions.

The calculations revealed an increase in ΔG_*comp*_ for **7** and a substantial stabilization for **8** and **9** (Table 1). Noteworthy, for both **8** and **9**, the most stable binding modes were found to be the coordination through two thiolates of Cys residues (Figure 2). No indications about the introduced residues partaking in Pb(II)‐coordination were noted, which could consume their capability of improving the overall solubility of the peptide in a complexed form.

The last parameter investigated was the further functionalization of the free carboxylic acid of γGlu. This modification would be synthetically feasible, as it allows for introducing new functional groups into the scaffold by coupling functionalized amines *via* amide bond formation post cyclization. Since in proteins, Pb(II) ion has an affinity for thiols and imidazole in ligands with mixed donors,^[26]^ cysteamine and histamine were chosen to be incorporated in peptides **10** and **11**, respectively. Congruent with the calculations for **4**, the enlarged side‐chains resulted in an increase in ΔG_*comp*_, which was about 30 and 40 kcal mol^−1^ for **10** and **11**, respectively. These changes likely result from unfavorable steric interactions between the relatively large opposing chains. Both modifications were therefore excluded for further investigation. We then synthesized peptides **2**, **3**, **5**, **8**, and **9** and tested their ability to recover Pb‐poisoned human cells *in vitro*.

### Peptide synthesis

Linear analogs of peptides **2**, **3**, **5**, **8**, and **9** were synthesized by standard Fmoc‐based solid‐phase peptide synthesis (SPPS) on 2‐chlorotrityl chloride resin, where the synthesis was initiated with Gly at the C‐terminus. The octapeptides were then cleaved off resin while being side‐chain protected,^[22]^ and were cyclized in solution where coupling conditions were adjusted to each precursor.^[22]^ Upon completion of cyclization,^[27]^ the peptides were side‐chain deprotected and further purified using HPLC.^[22]^

In addition, the linear analog of **2** (**lin2**) was also synthesized by cleaving it off the resin fully unprotected and purified by HPLC.^[22]^

### In vitro detoxification

**Figure 3:**
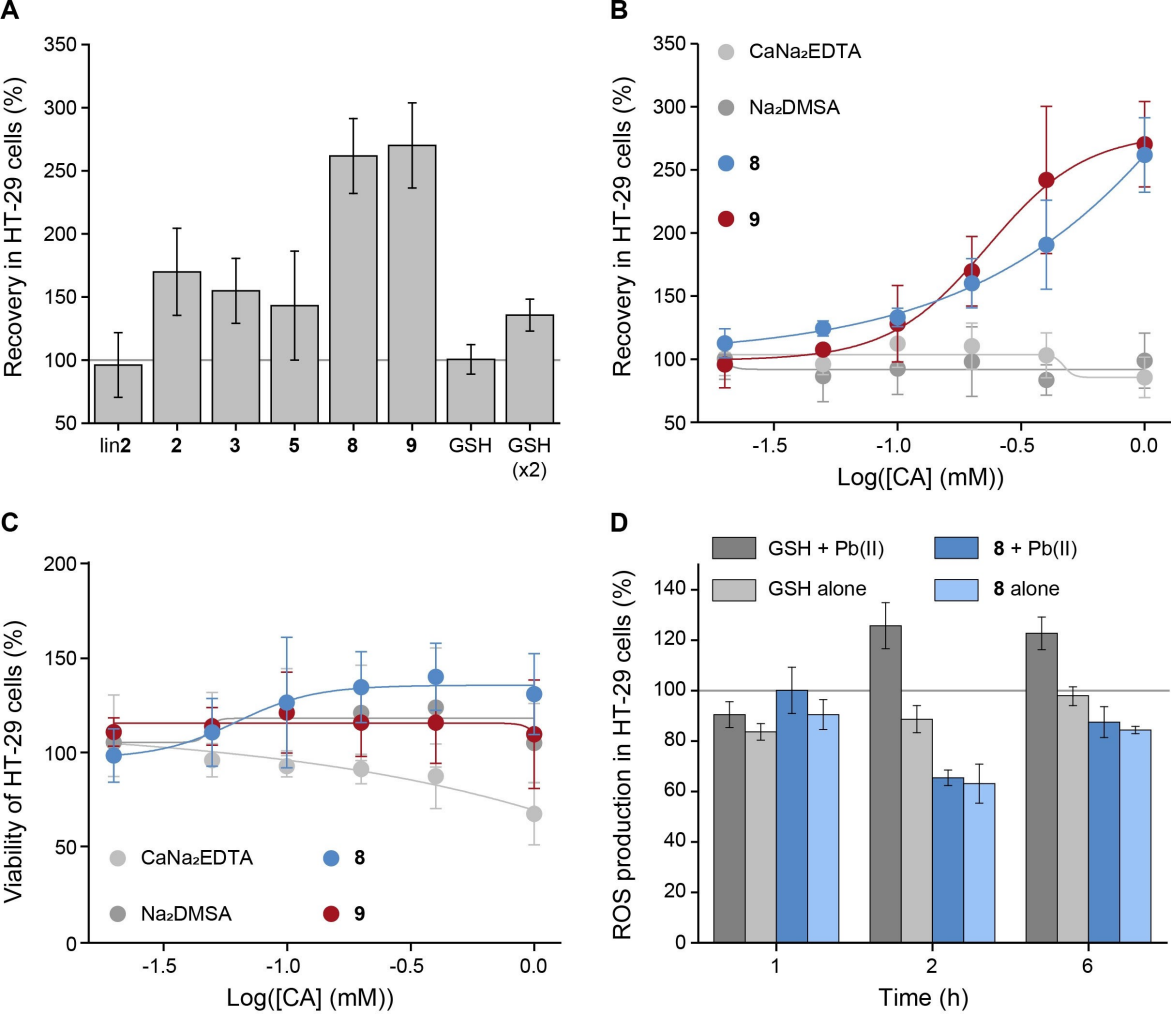
(A) Recovery (±SD) in HT‐29 cells after poisoning with Pb(NO_3_)_2_ (0.2 mM) followed by the administration of synthetic peptides including GSH at 1 mM (GSH at also 2 mM to reach the same equivalents of added thiols; 5 equiv.; 1 h after the addition of Pb(II) ions; values are calculated relative to cells poisoned with Pb(II) ions as the negative control), values from at least 3 independent experiments (B) dose‐dependent recovery (±SD) in HT‐29 cells poisoned with Pb(NO_3_)_2_ (0.2 mM) followed by the administration of **8**, **9** and benchmark drugs (1 h after the addition of Pb(II) ions; values are calculated relative to cells poisoned with Pb(II) ions as the negative control), values from at least 3 independent experiments, (C) dose‐dependent viability of HT‐29 cells treated with **8**, **9** and benchmark drugs, values from at least 3 independent experiments, (D) time‐dependent ROS production in HT‐29 cells in the presence of GSH or **8** (1 mM) administered with or without Pb(NO_3_)_2_ (0.2 mM; peptides were added 1 h after Pb(NO_3_)_2_; values are calculated relative to untreated cells as positive control).

Human HT‐29 cells were used to investigate the peptides′ detoxification capabilities.[^17^, ^18^, ^22^] Adhered cells were poisoned with Pb(NO_3_)_2_ (200 μM) and 1 h later treated with various concentrations of peptides^[28]^ and of the benchmark drugs, ranging from 20 μM to 1 mM that are equal to 0.1–5 equivalents compared with the Pb(II) concentration. After an incubation of 23 h, the cells were washed and quantified with the crystal violet assay.[^22^, ^29^] Each experiment included both positive and negative controls, which were either cells left untreated with both metal ions and peptides or only poisoned with Pb(NO_3_)_2_, respectively. Each dataset was then compared to the negative control to report the peptide recovery abilities (Figures 3A–B and S2).

Cyclization of **2** was revealed to be essential for detoxification, as reflected by the enhanced viability of cells treated with 1 mM of **2** compared with **lin2** (Figure 3A). The preorganization resulting from peptide cyclization is expected to enhance peptide stability and metal‐binding affinity, which is reflected in detoxification. These results reassured the benefits of cyclizing peptides for chelation.[^30^, ^31^]

Furthermore, treatment of the poisoned cells with all five cyclic peptides improved viability with increasing concentrations (Figures 3A, S2). Noteworthy, the recovery rates of the peptides align with their ΔG_*comp*_, apart from **5** that was expected to be a lead peptide based on the calculations but was found as the least active one. To achieve the desired efficiency, not only a high binding affinity, but also an enhanced metal selectivity is required.^[18]^ Due to its smaller macrocyclic cavity, we hypothesized that **5** might prioritize smaller metal ions such as Zn(II), Cu(II), etc.^[32]^ This would thus reduce the peptide's Pb selectivity. To examine this, ΔG_*comp*_ values for Zn(II)‐**5** and Cu(II)‐**5** were calculated (Table S2).^[22]^ The obtained values for **5** with Zn(II) and Cu(II) were more favorable (negative; −24.3 and −31.6 kcal mol^−1^, respectively) than for Pb(II)‐**5**, indicating a dramatically higher stability of complexation with physiologically occurring metal ions compared to Pb(II)‐**5**. The calculations thus corroborated the lack of efficacy identified *in vitro*.

Detoxification was most pronounced for **8** and **9**, reaching approximately 2.5 times the viability of the negative control (Figure 3A–B, Figure S2), demonstrating their ability to effectively detoxify Pb(II) ions. As expected, the polarity of the peptides seems to play a crucial role in their efficacy, which is reflected by the differences between **2** and the lead peptides **8** and **9**.

Significantly, all cyclic octapeptides, in particular, **8** and **9**, outperform the two drugs against Pb poisoning; DMSA and EDTA, and GSH, even when the tripeptide is administered in twofold excess compared with the octamers (Figures 3A–B, S2). These results, once again, corroborate the potential of peptides to act as chelating agents[^17^, ^18^] and of this scaffold specifically as a clever design that enables controlled complexation and detoxification.

We then tested the effect of **8** and **9** alone on cell viability compared with the two benchmark drugs. While, as expected, EDTA showed slight toxicity even at lower concentrations than have been examined before,^[17]^ **9** showed no toxicity at all, and **8** revealed to positively affect cell viability (Figures 3C, S3). We suspected that this enhanced viability might be due to an antioxidant activity of **8**. To test this, we performed the DCF assay[^33^, ^34^, ^35^] by which we treated HT‐29 cells with either GSH or **8** (1 mM), in the presence or absence of Pb(II) ions (0.2 mM; peptides were added 1 h after Pb(NO_3_)_2_; Figure 3D). The cells were washed extensively to remove any extracellular components, 1, 2, and 6 h after administering peptides.^[22]^ We then added dichlorodihydrofluorescein diacetate (H_2_DCF‐DA) as a fluorescent probe for reactive oxygen species (ROS).[^33^, ^34^, ^35^]

Comparing the fluorescence of the various conditions to untreated cells as a positive control reveals that 1 h after adding peptides under all conditions, ROS production is slightly below the baseline of 100 % (Figure 3D). After an additional 1 h, GSH with Pb(II) shows high levels of ROS production, reaching above 120 %, presumably since GSH is a weak binder of Pb(II). Hence, Pb(II) ions increase the cellular oxidative stress without interference. In contrast, adding **8** with or without Pb(II) decreased the oxidative stress compared to untreated cells, corroborating the antioxidative nature of **8**.

### Complex characterization

**Figure 4:**
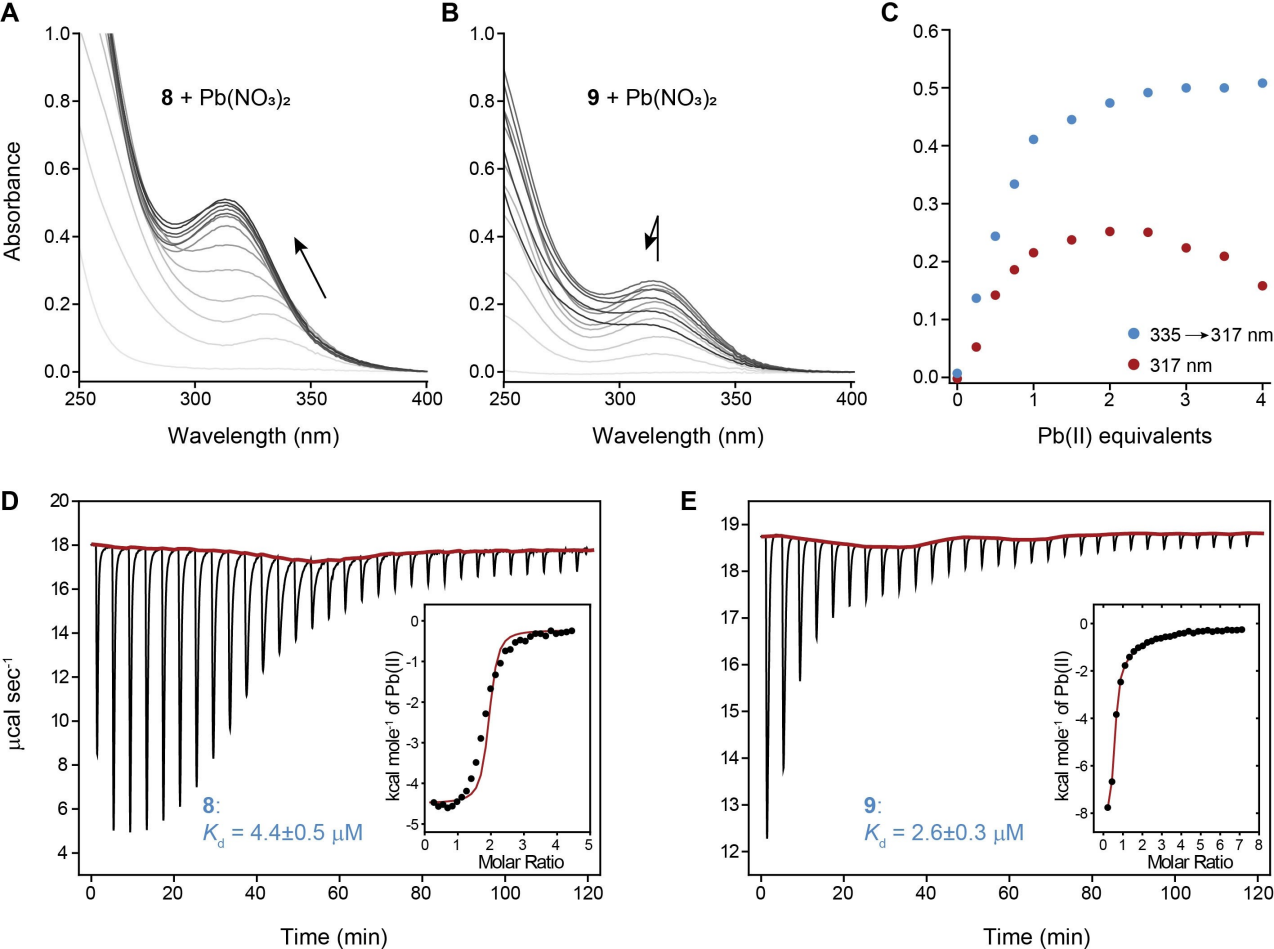
UV titration of **8** (A; 100 μM) and **9** (B; 100 μM) with Pb(NO_3_)_2_ (0–400 μM; 0–4 equiv.), where increased concentrations of Pb(II) are represented by the intensities of the gray lines, absorbance of **8** (blue) and **9** (red) titration with Pb(II) at 335→317 and 317 nm, respectively (C), ITC raw and integrated data for the titration of **8** (D), and **9** (E) with Pb(II) ions. Titrations were performed at 25 °C in 20 mM Tris buffer at pH 6.5.^[22]^ The titration curves were fitted to a one‐set‐of‐sites binding model using Origin software.

To further shed light on the detoxification capabilities of the lead peptides **8** and **9**, their complexation with Pb(II) ions was experimentally probed. UV titrations of **8** and **9** with Pb(NO_3_)_2_ were conducted to investigate the complex formation and determine their stoichiometries (Figure 4A–C). Therefore, the ligand‐to‐metal charge‐transfer (LMCT) bands were followed.

Titrations indicated the formation of Pb(II)‐peptide complexes at various ratios for both peptides. Adding Pb(NO_3_)_2_ (0–400 μM) to peptide **8** (100 μM) gave rise to a shoulder at 260 nm and a peak at λ_max_=335 nm. The shoulder and the peak were gradually blue‐shifted to 255 and 317 nm,[^23^, ^36^, ^37^] respectively, until a 1 : 1.5 ratio between **8** and Pb(NO_3_)_2_ was reached (Figure 4A, 4C). Absorption at 260 nm and LMCTs between 317–335 nm are attributed to S^−^ 3p→Pb^2+^ 6p LMCT and intraatomic Pb^2+^ 6 s→Pb^2+^ 6p transitions of Pb(II).[^23^, ^38^, ^39^, ^40^, ^41^] The plateau in the bands after 1.5–2 equivalents of added Pb(NO_3_)_2_ indicates a mixture of various Pb(II)‐**8** complexes with varied stoichiometries as 1 : 1 and 1 : 2. It also shows the participation of at least one of the thiols in Pb(II) coordination, as concluded in the computed lowest‐energy complex. HR‐ESI‐MS analysis of an equimolar mixture of **8** and Pb(NO_3_)_2_ corroborates the concluded stoichiometries by which the most dominant species is a homo‐binuclear complex, and the *apo* species is peripheral (Figure S4).

The titration of **9** with Pb(NO_3_)_2_ indicated the complexation at the same wavelengths as with **8** but without any maxima shift (Figure 4B–C). Unlike with **8**, after adding 2 equivalents of Pb(II) ions to **9**, the absorption started to decay. This could be due to the formation of Pb(II)‐**9** complexes that no longer have a similar print in the UV or due to aggregation.

We then determined the binding affinities of **8** and **9** to Pb(II) ions by isothermal titration calorimetry (ITC; Figure 4D–E).^[22]^ The dissociation constants (*K*_d_) are 4.4±0.5 and 2.6±0.3 μM for **8** and **9**, respectively. With an excellent alignment to the reported ΔG_*comp*_ values (Table 1), both peptides bind the toxic ion stronger than GSH.^[18]^

Depleting Zn(II) and Ca(II) ions as essential metals are frequently reported during chelation treatment for Pb‐poisoning and represent a significant drawback of the current CAs.[^9^, ^42^] Evaluating complex stability in the presence of such ions and metal selectivity is crucial in designing new CAs. We, therefore, proceeded to test these characteristics of **8** and **9** utilizing UV spectroscopy.

**Figure 5:**
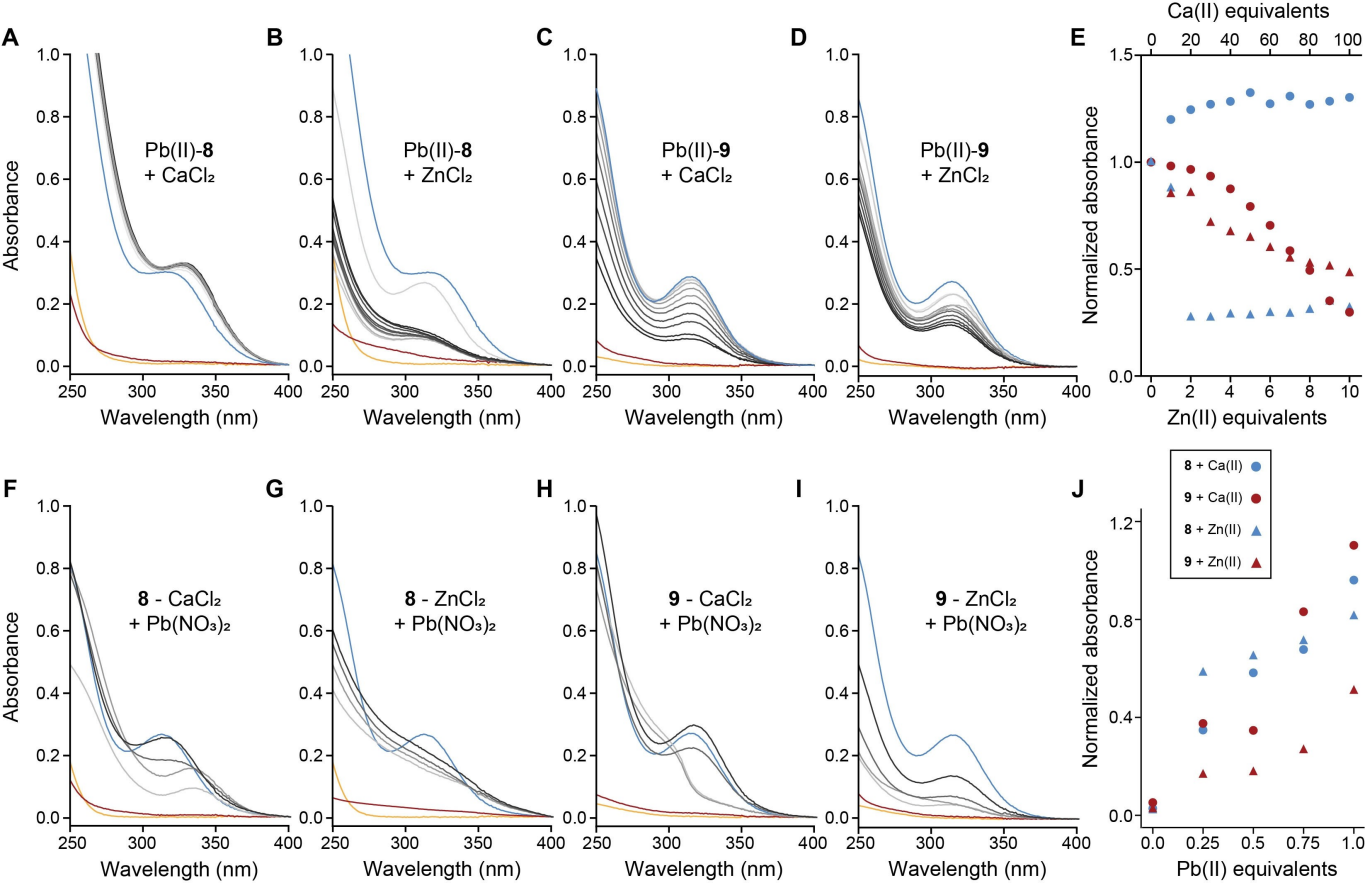
UV‐monitored back‐titrations of Pb(II)‐**8** (100 μM) with CaCl_2_ (A), ZnCl_2_ (B), Pb(II)‐**9** (100 μM) with CaCl_2_ (C), ZnCl_2_ (D), where the concentrations for CaCl_2_ are 0–10 mM (0–100 equiv.) and for ZnCl_2_ are 0–1 mM (0‐10 equiv.). Spectra of *apo* peptides are orange, peptides with competing ions in red, and *holo* in blue. Normalized absorbance (relative to *holo* without competitor) of **8** and **9** titrations with Ca(II) and Zn(II) at 335→317 and 317 nm, respectively (E), UV‐monitored back‐titrations of **8** and Ca(II) (10 mM; F), or Zn(II) (1 mM; G), **9** with Ca(II) (10 mM; H), or Zn(II) (1 mM; I) where peptides concentration is 100 μM with Pb(II) (0‐1 equiv.; 0–100 μM). Spectra of *apo* peptides are in orange, with competing ions in red and *holo* in blue. Normalized absorbance (relative to *holo* without competitor) of **8** and **9** mixed with Ca(II) and Zn(II) and titrated with Pb(II) at 335→317 and 317 nm, respectively (J).

To assess complex stability, equimolar mixtures of Pb(II) and peptides (100 μM) were back‐titrated with either ZnCl_2_ (0–1 mM; 0–10 equiv.) or CaCl_2_ (0–10 mM; 0–100 equiv.), and the stability was assessed by following the LMCT bands at 317–335 nm. Titrating CaCl_2_ to Pb(II)‐**8** shifted the LMCT band from 317 nm to 330 nm but did not lower its intensity, indicating the stability of the complex in the presence of Ca(II) ions (Figure 5A, 5E). Adding an equimolar concentration of ZnCl_2_ to the complex also did not significantly affect the LMCT band (Figure 5B). However, after a second equivalent was added, the absorption of the LMCT band dropped to around 30 % and stayed relatively constant after the addition of further equivalents (Figure 5B, 5E). Therefore, ZnCl_2_ seems to disrupt the integrity of the Pb(II)‐**8** complex. Noteworthy, no direct evidence of a Zn(II)‐**8** complex was observed, as the absorption spectrum of a mixture containing ZnCl_2_ and **8** (10 : 1) did not show any additional features compared to the spectrum of *apo* **8** (Figure 5B). A HR‐ESI‐MS analysis of an equimolar mixture of **8** with ZnCl_2_ corroborates that no complexation with the essential ion occurs, and the vast majority of the peptide persists at its *apo* state (Figure S5).

To further understand these observations, we examined by HR‐ESI‐MS three mixtures of **8** with both Pb(NO_3_)_2_ and ZnCl_2_, all with an equimolar ratio of peptide and Pb(II) ions and differed in the concentrations of Zn(II) ions; 1, 3, and 5 equivalents (Figures S6–8). When adding 1 equivalent of Zn(II) ions, the most dominant species were the homo‐binuclear complex with Pb(II) ions only and the hetero‐binuclear complex, where only minuscule amounts of Zn(II)‐**8** were detected (Figure S6). Adding 3 or 5 equivalents of Zn(II) ions increased the ratio between the hetero‐binuclear Pb(II)‐Zn(II)‐**8** and the Pb(II)_2_‐**8** complexes, indicating that Zn(II) could displace one of the Pb(II) ions from the binuclear Pb(II)_2_‐**8** species (Figures S7–8). This hints that the decrease in the LMCT as a response of Zn(II) ions addition is not a result of a complete ion replacement but the formation of a different complex with Pb(II) ion.

In the case of **9**, the titration with both CaCl_2_ and ZnCl_2_ resulted in a steady decrease of the absorption at 317 nm to 29 % and 48 % of *holo* **9**, respectively, at the highest concentrations of the essential metals (Figure 5C–E). Therefore, the complex did not indicate high stability in the presence of either of the essential metals, revealing the inferiority of **9** over **8**.

To learn more about the selectivity of these peptides, we performed reversed back‐titrations (Figure 5F–J), adding up to one equivalent of Pb(NO_3_)_2_ compared to the peptide to mixtures of peptides and CaCl_2_ or ZnCl_2_ in molar ratios of 1 : 100 and 1 : 10, respectively. **8** showed the same behavior when the CaCl_2_‐containing mixture was titrated as in the titration without Ca(II) ions (Figure 5F, 5J). However, when titrating Pb(II) to a mixture of **8** and ZnCl_2_, the spectra showed no typical LMCT at 317–335 nm, but the overall absorbance was increased as a response to Pb(II) addition (Figure 5G). Looking at the spectra of both opposite titrations and based on the calculated lowest‐energy complex of Pb(II) with **8**, we hypothesize that in the absence of Zn(II) ions, Pb(II) binds to both thiols of **8**, as detected in the UV spectrum and with an agreement with calculations. Once Zn(II) is added, **8** binds Pb(II) via the two carboxylates, which is the second lowest‐energy complex according to calculations, with ΔG_*comp*_=−5.3 kcal ⋅mol^−1^, only 0.8 units weaker than the thiol‐based binding mode. With such coordination, **8** could bind Zn(II) ion via the thiolates without losing the toxic ion. Additionally, mixed coordination environments would also be possible. In fact, HR‐ESI‐MS spectra of **8** with Pb(II) and excess of Zn(II) (Figures S7–8) show that the most dominant species is, indeed, the hetero‐binuclear complex. Such a complex can explain the disappearance of the clear LMCT at 317–335 nm.

The reversed back titrations of **9** with CaCl_2_ indicated a complete complexation with Pb(II) ions, even when the former was in a 100 fold excess and Pb(II) in an only equimolar ratio (Figure 5H, 5J). When adding 0.25 and 0.5 equivalents of Pb(II) ions compared with **9**, the LMCT was red‐shifted and appeared as a shoulder. Presumably, this occurred due to the increased number of coordinating thiols,^[36]^ which could result from Ca(II) ions impacting the coordination of the ligand to Pb(II). After adding more Pb(II) ions, the spectra appeared similar to the *holo* in the absence of competing ions. The presence of 10 equivalents ZnCl_2_ did not affect the spectrum shape upon adding Pb(II) ions, but the overall intensity reached only 42 % of the *holo* absorption.

The analytical investigations showed that both peptides **8** and **9** could form complexes with Pb(II) ions, even in the presence of significant excess of essential metal ions. Peptide **8** formed a complex with Pb(II) that was superior in stability to Pb(II)‐**9** in the presence of Ca(II) and Zn(II) ions. We were, therefore, encouraged to continue further with our investigation with **8**.

### Mode of action

In order to determine the mode of action of **8**, by which it enables to achieve a high recovery of Pb‐poisoned HT‐29 cells, inductively coupled plasma mass spectrometry (ICP‐MS) was employed to determine the Pb content of the Pb‐poisoned cells and their respective supernatant. First, HT‐29 cells were poisoned with Pb(NO_3_)_2_ (0.2 mM) and the Pb content in the cells and in the medium was determined after 1 and 24 h.^[22]^ The measurement after 1 h was performed to assess the Pb distribution at the time of peptide addition during the recovery experiment and the one after 24 h to reveal the distribution resulting without intervention. At both times, the fraction of intracellular Pb was low and most of the toxic metal was in the medium (Table S10). This indicates either that a low intracellular Pb concentration is sufficient to cause significantly reduced viability or that toxicity in this model can occur *via* an extracellular mechanism such as membrane disruption. Next, we added **8** to the cells before and after poisoning them with Pb(II) ions to assess whether **8** prevents Pb(II) ions from entering the cells, expels them from the cells or transforms Pb(II) into a non‐toxic complex locally. The results clearly show that when **8** is added to cells pre‐ or post‐poisoning, the supernatant‐to‐cells ratio of detected Pb is reduced by approximately half compared to cells that were not treated with the peptide. Since treating Pb‐poisoned cells with **8** enables higher viability, despite the fact that their relative intracellular Pb content is elevated, we hypothesize that Pb crosses the cellular membrane from outside as a membrane permeable, non‐toxic Pb(II)‐**8** complex.

### Peptide stability

To further examine the potential of **8** as a next‐generation chelating agent against Pb‐poisoning, we determined the oxidation rate of the peptide at which it forms a disulfide bond. Since all peptides within this scaffold contain two Cys residues that are oriented towards each other, intramolecular oxidation is plausible to occur spontaneously since no significant entropic penalty is apparent.

To examine this, we used the Ellman's test,^[43]^ where **8** was incubated in H_2_O, and the Ellman's reagent was added at given time points (Figure S9).^[22]^ While **8** was started to be slightly oxidized within the first 2 h and was steadily forming disulfide bonds, after 24 h, roughly 40 % of the thiols were still reduced, enabling metal coordination within the experimental time frame of our *in vitro* study. Furthermore, **8** was found to be stable in human blood serum and at 37 °C for at least 48 h (Figure S10), revealing, once again, the advantages of cyclization.

## Conclusions

With the aid of state‐of‐the‐art computational tools, we rationally designed and investigated a peptidic scaffold that contains two units of GSH and a total of eight AAs. Upon sequence optimization based on computational scoring of eleven peptides and *in vitro* evaluations of five members, the two lead peptides revealed an outstanding capability to recover Pb‐poisoned human cells of up to 2.5 folds compared with untreated cells outcompeting the standards of care as well as GSH. The peptides were also found to be non‐toxic, one of which showed antioxidant activity as it also enhanced cell viability when administered alone. Noteworthy, the utilized computational tools were capable, in general, of predicting complex stability, which is correlated to the bioactivity of the examined peptides. The calculations proved efficient in prioritizing plausible candidates within the scaffold.

The lead peptides were found to be highly selective for Pb(II) ions over Ca(II), and even though the presence of Zn(II) ions changed the complexation with Pb(II) of one of the peptides, it still captures the toxic ion. The increase in peptide efficacy compared to GSH is beyond an additive improvement, indicating the contribution of three parameters; cyclization, cavity size, and polarity. All of these enhance metal affinity and selectivity that are then translated into the detoxification of Pb(II) ions *in vitro*.

## Conflict of interest

The authors declare no conflict of interest.

1

## Supporting information

As a service to our authors and readers, this journal provides supporting information supplied by the authors. Such materials are peer reviewed and may be re‐organized for online delivery, but are not copy‐edited or typeset. Technical support issues arising from supporting information (other than missing files) should be addressed to the authors.

Supporting Information

## Data Availability

The data that support the findings of this study are available in the supplementary material of this article.
