# Supplementary material for: Cyclic Octapeptides Composed of Two Glutathione Units Outperform the Monomer in Lead Detoxification
Source: ChemMedChem. 2022 May 24;17(15):e202200152. doi: 10.1002/cmdc.202200152 (PMC9544108; doi:10.1002/cmdc.202200152)
Supplement: Supplementary file 1 — Supporting Information [file CMDC-17-0-s001.pdf]

ChemMedChem

Supporting Information

Cyclic Octapeptides Composed of Two Glutathione Units Outperform the Monomer in Lead Detoxification

Luca Sauser, Tadeáš Kalvoda, Ayça Kavas, Lubomír Rulíšek, and Michal S. Shoshan*

Table of Contents

1	General materials and methods	2
2	Quantum chemical calculations	4
3	General protocols for SPPS	8
4	Analytical data of the peptides	12
5	*In vitro* studies	15
6	Complexes characterization	19
7	Ellman's test	26
8	Mode of action	28
9	Stability in HBS	30
10	References	31

1 General materials and methods

Materials and reagents were of the highest grade available, purchased from ABCR, Sigma Aldrich, TCI, or Fluorochem, and used without further purification. Amino acids were purchased from Merck, Bachem, Novabiochem, Senn, or ABCR and used as received. Water used for peptide preparation and purification was nanopure ("Milli-Q") prepared by a Barnstead GenPure system (ThermoFisher Scientific).

Solid-phase peptide synthesis was performed on 200–400 mesh 2-Chlorotrityl chloride polystyrene resin from ABCR.

UPLC-ESI-MS measurements were performed on Waters Acquity UPLC system coupled to a Bruker Daltonics HCTTM ESI-MS, using an Acquity UPLC BEH C18 1.7 µm (2.1 x 50 mm) column. UPLC solvents were formic acid (0.1% in Millipore H_2O) and CH_3CN UPLC grade. Applied UPLC gradient: 0–0.5 min: 5% CH_3CN; 0.5–4.0 min: gradient from 5% to 100% CH_3CN; 4.0–5.0 min: 100% CH_3CN at 40 °C. The flow rate was 0.6 mL min^{-1}. Detection was performed by UV absorption from 200-400 nm.

HPLC purification was performed on Dionex UltiMate 3000 UHPLC system, using a Reprosil Gold C18 120 (250 x 20 mm) column. Solvents were 0.1% TFA in H_2O and CH_3CN at various temperatures and gradients, as described in Table S3.

^{1}H and ^{13}C NMR spectra were recorded on a Bruker AVII-400 (400 MHz) or Oxford NMR AS 500 (500 MHz), chemical shifts are reported in ppm using the solvent residual peak as reference (D_2O 4.79 ppm for ^{1}H).

High-resolution mass spectrometry measurements were performed on a timsTOF Pro TIMS-QTOF-MS instrument (Bruker Daltonics GmbH).

Cell culture Human colon adenocarcinoma cells (HT-29) were purchased from the American Type Culture Collection (ATCC). The cells were grown in a humidified 5% CO_2 atmosphere at 37 °C using Roswell Park Memorial Institute medium (RPMI-1640) with 25 mM HEPES, supplemented with 1% L-glutamine, 1% penicillin/streptomycin, and 10% fetal calf serum (FCS) superior (standardized). Culture medium RPMI-1640-HEPES (with L-glutamine), penicillin (10.000 U/mL), and streptomycin (10 mg/mL) were purchased from Sigma Aldrich, Invitrogen, or BioConcept. Trypsin-EDTA (0.02%) in Ca(II) and Mg(II) deficient phosphate-buffered saline (PBS) (1:250) was

purchased from Amimed. FCS superior was purchased from Oxoid AG and Biochrom AG. Crystal violet (CV) was purchased from Sigma Aldrich. *In vitro* assays were monitored on a Hidex Sense microplate reader.

UV-Vis spectrophotometer selectivity and stability measurements were performed on a Lambda 850 UV-Vis spectrophotometer (PerkinElmer) with an ultra-micro Suprasil quartz cuvette (Hellma) with a chamber volume of 100 μL and a path length of 10 mm. Wavelength scans were performed at 25 °C from 200 to 400 nm, with a 40 nm min^{-1} scan speed.

Isothermal titration calorimetry (ITC) experiments were performed on a VP-ITC MicroCalorimeter (MicroCal, Malvern) at 25 °C. The ITC data were analyzed using the Origin software and fitted by one or two sets of sites binding models.

High-resolution mass spectrometry was performed on a *Dionex Ultimate 3000* UHPLC system (ThermoFischer Scientifics, Germering, Germany) connected to a QExactive MS with a heated ESI source (ThermoFisher Scientific, Bremen, Germany); onflow injection of 1 μL sample (c = ca. 50 μg mL^{-1} in the indicated solvent) with an XRS auto-sampler (CTC, Zwingen, Switzerland); flow rate 120 μL min^{-1}; ESI: spray voltage 3.0 kV, capillary temperature 280 °C, sheath gas 30 L min^{-1}, aux gas 8 L min^{-1}, s-lens RF level 55.0, aux gas temperature 250 °C (N2); full scan MS in the alternating (+)/(−)-ESI mode; mass ranges 80–1’200 m/z, 133–2’000 m/z, or 200–3’000 m/z at 70’000 resolution (full-width half-maximum); automatic gain control (AGC) target of 3.00·106; maximum allowed ion transfer time (IT) 30 ms; mass calibration to <2 ppm accuracy with Pierce® ESI calibration solns. (ThermoFisher Scientific, Rockford, USA); lock masses: ubiquitous erucamide (m/z 338.34174, (+)-ESI) and palmitic acid (m/z 255.23295, (−)-ESI).

ICP-MS measurements were performed with an Agilent QQQ 8800 Triple quad ICP-MS spectrometer, equipped with a standard x-lens setting, nickel cones and a “micro-mist” quartz nebulizer. The feed was 0.1 ml/min, the RF power 1550 W. Tune settings were based on the Agilent General Purpose method and only slightly modified by an autotune procedure using an Agilent 1 ppb tuning solution containing Li, Y, Ce and Tl. Values are reported as the average of 30 sweeps x 3 replicates. Elements were measured in a “helium-mode”. The name is referring to the gas in the reaction cell. All solutions were prepared from 60% HNO_3 (Merck 1.01518.1000 ultrapure), 30% HCl (Merck 1.01514.1000 ultrapure), or aqua regia (1:3 mixture of 60% HNO_3 and

30% HCl 1:3, ultrapure) and 18.2 MΩ Millipore water. Elements were measured against a serial dilution made with the following standard: Lutetium: Merck 1.70330.0100 in 2% HNO_3, was used as internal standard.

2 Quantum chemical calculations

Computational details

All DFT calculations were carried out using the Turbomole 7.5.1 program.[1] Geometries of all structures were optimized employing the dispersion-corrected BP86 functional [BP86-D3(BJ)][2–5] and the def2-TZVP[6] basis set (for Pb, this basis set includes the Stuttgart-Dresden effective core potentials) in an implicit solvent with the dielectric constant of ε_r = 80 corresponding to water - employing the COSMO solvation model[7] as implemented in Turbomole 7.5.1 program. Subsequent vibrational frequency calculations were performed at the same level of theory for all calculated structures in vacuo after reoptimization (in vacuo). All equilibrium structures had all Hessian eigenvalues positive, so all stationary points were confirmed to be genuine minima on the potential energy surface (PES).

According to previous work,[8] the free energy value corresponding to a particular structure/molecule can be conveniently expressed as (Equation S1):

$$G_S = E_{el} + \Delta G_{solv} + E_{ZPVE} - RTln(q_{trans}q_{rot}q_{vib}) + pV \qquad \text{(S1)}$$

where E_{el} is the electrostatic potential energy of the molecule *in vacuo* (gas-phase molecular energy), calculated with def2-TZVPD basis set, and the functional BP86-D3(BJ), ΔG_{solv} is the solvation energy calculated by employing the COSMO-RS method (*vide infra*), E_{ZPVE} is the zero-point vibrational energy whereas $RT\ln(q_{trans}q_{rot}q_{vib})$ are the entropic terms obtained from the rigid-rotor/harmonic oscillator (RRHO) approximation in which a free rotor model was applied for low-lying vibrational modes under 100 cm^{-1} with a smoothing function applied (sometimes denoted as quasi-RRHO, or RRFRHO approximation). The E_{el} and (E_{ZPVE} - $RT\ln(q_{trans}q_{rot}q_{vib})$ + pV) are calculated for the *in vacuo* equilibrium structures. For all structures, we checked that the *in vacuo* minima do not significantly deviate from their solvent counterparts.

The ΔG_{solv} was obtained using Klamt's conductor-like screening model for the realistic solvation method (COSMO-RS).[9] COSMO-RS calculations were carried out using *cosmotherm21* software with the parameter file "BP_TZVPD_FINE_C30_2101.ctd" and the recommended protocol: BP86-D3(BJ)/def2-TZVPD single point calculations *in vacuo* (on top of the *in vacuo* geometries) and in an ideal conductor ($\varepsilon = \infty$) for the solvent geometry, followed by the COSMO-RS (*cosmotherm21*) calculations in the target solvent (water). Throughout, FINE cavities ($cosmo_isorad keyword) were used to increase numerical precision. Finally, a correction of ($1.9\cdot\Delta n$) kcal mol^{-1} (corresponding to the difference between the concentration of the ideal gas at 298 K and 1 atm and its 1 mol L^{-1} concentration; Δn is the change in the number of moles in the reaction) has been applied in order that the computed values refer to 1 mol L^{-1} standard state.

Definition of a complexation energy

The following definition of the complexation Gibbs free energy, ΔG_{comp} (which relates to K_d as $\Delta G_{comp} = RT\ln K_d$) has been used throughout (Equation S2):

$$\Delta G_{comp} = G_S(products) - G_S(reactants) \tag{S2}$$

The "reaction" used is the following (Equation S3):

$$[Pb(H_2O)_5] + \mathbf{X(\text{-}}q\mathbf{H)} \rightarrow [Pb\text{-}\mathbf{X}^{(q-)}\cdot(H_2O)_m]\cdot n\{H_2O\}) + (5 - m - n)\ H_2O + q\ H^+ \tag{S3}$$

where 5 is the optimal hydration number for Pb(II) (the lone pair on Pb(II) leads to the hemidirected hydration and the $n = 5$ to be the optimal coordination number); m denotes the number of the first-sphere, n the number of second-sphere waters, and q the number of protons dissociated upon Pb(II) binding.

Table S1. Number of water molecules (*m* + *n*) for studied peptides **1-11** and a number of released protons (*q*)

**Peptide**	***m+n***	***q***
1	4	2
2	4	2
3	4	2
4	4	1
5	4	1[a]
6	4	2
7	4	2
8	2	2
9	4	2
10	4	1
11	4	2

[a] q = 0 for complexation with Zn(II)

This approach was previously tested[8] and compared with an alternative approach involving deprotonated (thiol) groups already on the l.h.s. of the Equation S3 and the experimental/computed pK_a values of thiol groups. Both led to similar results, which proves the robustness of the used computational protocol.

To find the global minimum of the peptide **X** in solution, we employed the Maestro/MD-LLMOD program (Schrodinger, Inc.) with default settings to obtain the initial set of ~200 conformers for each peptide, which were further processed by the QM//COSMO-RS protocol described above. Thus, we expect that we located the global minimum for each peptide **X**.

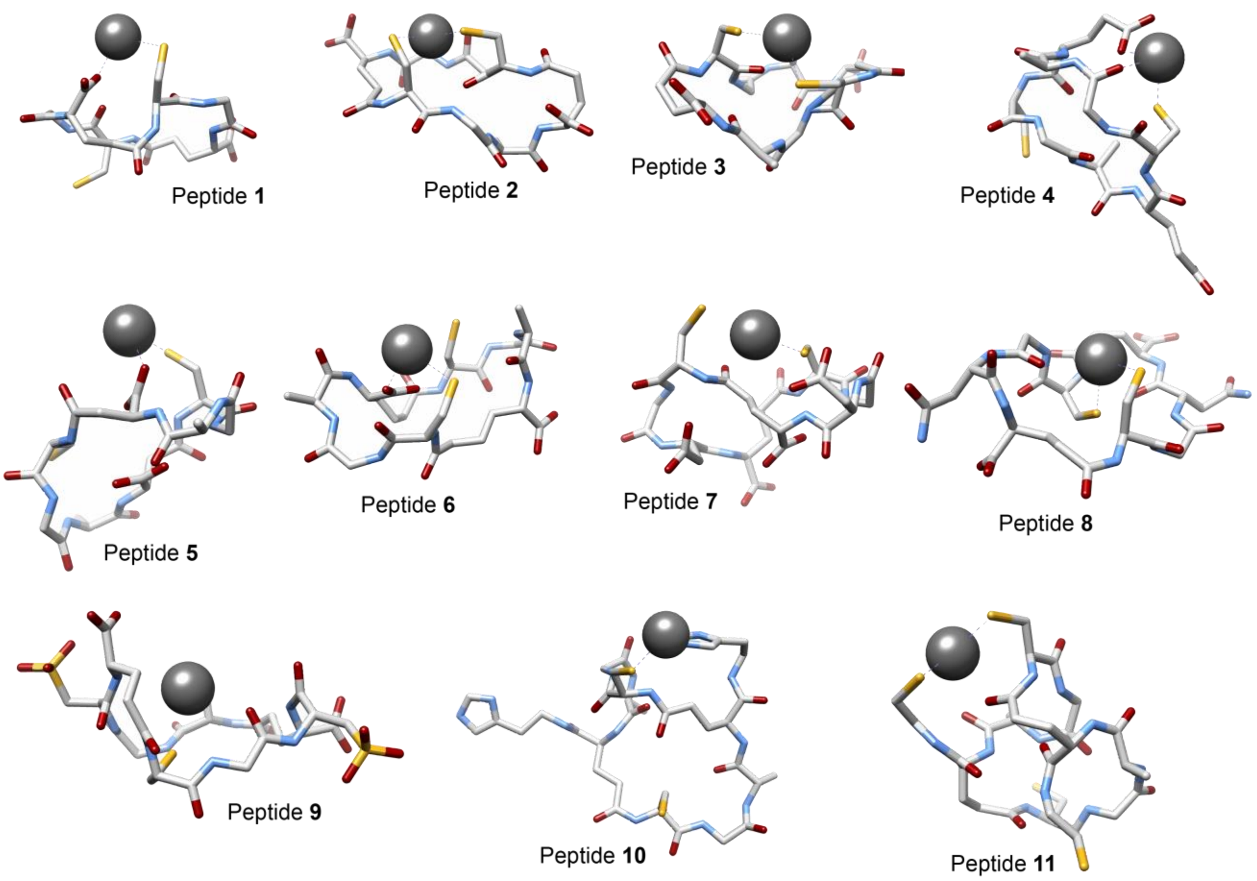

Figure S1. Calculated lowest-energy structures of [Pb-**X**·$(H_2O)_m$]·n{H_2O} where X is all eleven examined peptides and m + n = 4. Hydrogen atoms and four water molecules, placed in the first or the second coordination spheres, were omitted for clarity.

Calculations of complexation of Zn(II) and Cu(II) by peptide 5

The calculations of the complexation Gibbs free energy was carried out in the same manner like in the case of Pb(**II**). As a reference state, we used the hexahydrate of Zn(**II**) and pentahydrate of Cu(**II**), so n = 6 for Zn(**II**) and n = 5 for Cu(**II**) in equation S3. In both cases, four water molecules were used as a model of the first hydration sphere (m+n = 4). In case of Zn(**II**), no protons were released during the complexation (q = 0); one proton was released during the complexation of Cu(**II**) (q = 1).

Table S2. Computed complexation Gibbs free energy values for the complexation of peptide **5** with Zn(II) and Cu(II)

**Metal ion**	**ΔG_{comp} (kcal mol^{-1})**
Zn(II)	-24.3
Cu(II)	-31.6

3 General protocols for SPPS

3.1 Coupling of the first amino acid

The resin was washed with dry CH_2Cl_2, and a mixture of Fmoc-Xaa-OH (3 equiv.) and Hünig's base (6 equiv.) in dry CH_2Cl_2 was added. The resin was agitated for 2 h and then washed with DMF (x3) and CH_2Cl_2 (x3).

3.2 Fmoc deprotection

A solution of 20% piperidine in DMF was added to the resin, and the suspension was agitated for 10 min. The procedure was repeated twice. The resin was then washed with DMF (x3) and CH_2Cl_2 (x3).

3.3 Amino acid couplings

Fmoc-Xaa-OH (3 equiv.) and HATU (2.7 equiv.) were dissolved in DMF (0.8 M), and Hünig's base (6 equiv.) was added. After 1 min, the mixture was added to the amino-functionalized resin that was suspended in a minimal amount of DMF. The mixture was agitated for 60 min and washed with DMF (x3) and CH_2Cl_2 (x3). Couplings were monitored by test-cleavage of a small portion of resin and subsequent LCMS analysis.

3.3.1 Coupling of Fmoc-Cya-OH

Fmoc-Cya-OH (1.5 equiv.) and HATU (1.35 equiv.) were dissolved in DMF:DMSO (1:1) and Hünig's base (3 equiv.) was added. The mixture was added to the amino-functionalized resin agitated for 4 h. Afterward, the resin was washed with DMF:DMSO (1:1; x3) and CH_2Cl_2 (x3). Couplings were monitored by test-cleavage of a small portion of resin and subsequent LCMS analysis.

3.4 Cleavage and side-chain deprotection from the solid support

The resin was agitated with the mixture TFA:H_2O:TIPS:EDT (87.5:2.5:2.5:7.5; 3x30 min) and washed with CH_2Cl_2 (x3). The combined solution was collected by filtration and concentrated under a stream of nitrogen, and the product was precipitated from cold Et_2O. The white solid was isolated by centrifuging the suspension and decanting the supernatant. The solid was triturated with Et_2O (x2) and CH_3CN (x3). The residual white solid was dried under a stream of nitrogen, dissolved in H_2O, and lyophilized.

3.5 Cleavage of side-chain protected peptides from the solid support

The resin was agitated with a solution of 20% HFIP in CH_2Cl_2 (3x15 min) and then washed with CH_2Cl_2 (x3). The combined solution was collected by filtration and concentrated under reduced pressure. The product was subsequently precipitated with cold Et_2O, and the precipitate was isolated by centrifuging the mixture and collecting the resulting pellet. The pellet was washed twice with cold Et_2O, redissolved in H_2O:CH_3CN (1:1), and lyophilized.

3.6 Head-to-tail cyclization of side-chain protected linear peptides

The protocol was adjusted for each sequence, as follows:

2: to side-chain protected **lin2** (1 mM) in CH_2Cl_2, HATU (1.5 equiv.) and Hünig's base (1.7 equiv.) were added. The mixture was stirred at RT for 14 d. As LCMS analysis did not reveal full conversion or indicate further reaction progression, the solvent was removed under reduced pressure. The brown solid was resuspended in H_2O:CH_3CN (1:1) and lyophilized.

3 and **8**: to side-chain protected **lin3** or **lin8** (1 mM) in CH_2Cl_2, HATU (1.5 equiv.) and Hünig's base (1.7 equiv.) were added. The mixture was stirred at RT for 24 h. LCMS analysis indicated full conversion of the starting material. The solvent was removed under reduced pressure, and the brown solid was resuspended in H_2O:CH_3CN (1:1) and lyophilized.

5: to side-chain protected **lin5** (1 mM) in CH_2Cl_2, PyBOP (1.5 equiv.) and Hünig's base (1.7 equiv.) were added. The mixture was stirred at RT for 7 d. As LCMS analysis did not reveal full conversion or indicate further reaction progression, the solvent was removed under reduced pressure. The brown solid was resuspended in H_2O and lyophilized.

9: to side-chain protected **lin9** (1 mM) in CH_2Cl_2, HATU (1.5 equiv.) and Hünig's base (1.7 equiv.) were added. The mixture was stirred at RT for 48 h. LCMS analysis indicated full conversion of the starting material. The solvent was removed under reduced pressure after this period. The brown solid was resuspended in H_2O and lyophilized.

3.7 Side-chain deprotection of cyclic peptides

A deprotection mixture (TFA:H_2O:TIPS:EDT; 88:2.5:2.5:7; 30 mL g^{-1}) was added to the crude lyophilized mixtures from the cyclization, and the solution was stirred at RT for 3 h. The solution was concentrated under a stream of nitrogen. The product was precipitated with cold Et_2O, and the precipitate was isolated by centrifuging the mixture and collecting the resulting pellet. The pellet was washed twice with cold Et_2O, redissolved in H_2O:CH_3CN (1:1), and lyophilized.

3.8 Peptide purifications by HPLC

Crude peptides were dissolved in H_2O. The solutions were centrifuged to get rid of undissolved constituents and then filtered. Solutions were then injected into HPLC using 0.1% TFA in H_2O and CH_3CN as eluent system. The collected fractions were lyophilized, and their purity was determined by LCMS, HR-ESI-MS, and NMR spectroscopy.

Table S3 HPLC conditions for peptide purifications

	Step 1	Step 2	Temp. (°C)	Rt (min)
**lin2**	2 min, 5% CH_3CN	15 min, 5 to 10% CH_3CN	RT	8.7
**2**	2 min, 5% CH_3CN	15 min, 5 to 20% CH_3CN	50	9.8
**3**	2 min, 16.5% CH_3CN	15 min, 16.5 to 17% CH_3CN	50	10.5
**5**	2 min, 14% CH_3CN	15 min, 14 to 19% CH_3CN	35	12.0
**8**	2 min, 12.5% CH_3CN	25 min, 12.5 to 15% CH_3CN	50	9.5
**9**	2 min, 7% CH_3CN	15 min, 7 to 10% CH_3CN	30	9.8

3.9 Ion-exchange

Purified peptides (~10 mg) were reacted with HCl (32%, ~1 mL) for 1 min, and the solvent was removed under reduced pressure. The procedure was repeated at least two more times until no TFA signal was observed by recording ^{19}F NMR spectra.

4 Analytical data of the peptides

4.1 H_2N-[Ala-γGlu-Cys-Gly]$_2$-OH (**lin2**)

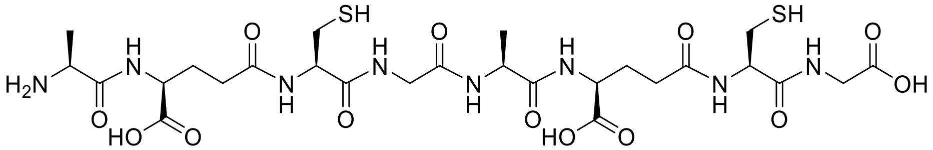

^{1}H NMR (400 MHz, D_2O) δ (ppm) 4.57 (t, *J* = 5.5 Hz, 1H), 4.52 (t, *J* = 6.6 Hz, 1H) 4.45 – 4.31 (m, 3H), 4.13 (q, *J* = 7.3 Hz, 1H), 3.99 (d, J = 19.7 Hz, 4H), 2.97 – 2.86 (m, 4H), 2.48 (t, *J* = 7.3 Hz, 4H), 2.33 – 2.19 (m, 2H), 2.09 – 1.95 (m, 2H), 1.55 (d, *J* = 7.1 Hz, 3H), 1.39 (d, *J* = 8.0 Hz, 3H)

^{13}C NMR (101 MHz, D_2O) δ (ppm) 175.5, 175.4, 175.3, 175.2, 174.8, 173.5, 172.7, 172.3, 170.9, 170.6, 55.8, 55.5, 53.1, 52.3, 49.6, 48.9, 42.4, 41.5, 31.5, 31.4, 26.6, 26.5, 25.5, 25.2, 16.5, 16.4

HRMS (ESI) *m/z* calculated for $C_{26}H_{43}N_8O_{13}S_2^+$ [M+H]$^+$ 739.23855; found: 739.23886

LCMS (ESI) Rt = 1.0 min, observed as [M+H]$^+$

4.2 *cyc*[Ala-γGlu-Cys-Gly]$_2$ (**2**)

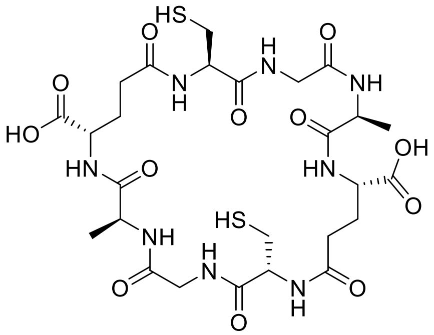

^{1}H NMR (400 MHz, D_2O) δ (ppm) 4.68 – 4.61 (m, 2H), 4.40 – 4.28 (m, 4H), 4.00 (dd, *J* = 23.1 Hz, 4H), 2.91 (d, *J* = 6.1 Hz, 4H), 2.48 – 2.41 (m, 4H), 2.38 – 2.26 (m, 2H), 2.02 – 1.90 (m, 2H), 1.39 (d, *J* = 7.2 Hz, 6H)

^{13}C NMR (126 MHz, D_2O) δ (ppm) 175.4, 175.2, 174.7, 172.4, 170.7, 55.4, 51.9, 49.6, 42.2, 31.4, 26.7, 25.7, 16.03

HRMS (ESI) *m/z*: calculated for $C_{26}H_{41}N_8O_{12}S_2^+$ (M+H)$^+$ 721.22799; found: 721.22698

LCMS (ESI) Rt = 1.2 min, observed as [M+H]$^+$

4.3 *cyc*[Ala-γGlu-cys-Gly-Ala-γGlu-Cys-Gly] (**3**)

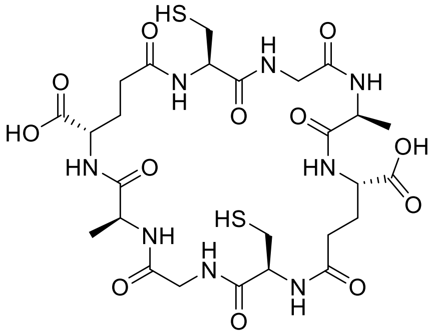

¹H NMR (400 MHz, D_2O) δ (ppm) 4.53 (q, *J* = 6.0 Hz, 2H), 4.41 – 4.31 (m, 4H), 4.01 – 3.86 (m, 4H), 2.94 (t, *J* = 5.7 Hz, 4H), 2.47 (q, *J* = 7.1 Hz, 4H), 2.38 – 2.25 (m, 2H), 2.05 – 1.88 (m, 2H), 1.40 (d, *J* = 4.0 Hz, 3H), 1.38 (d, *J* = 4.1 Hz, 3H)

¹³C NMR (126 MHz, D_2O) δ (ppm) 175.5, 175.4, 175.3, 175.2, 174.9, 174.7, 172.8, 172.6, 170.9, 170.8, 55.8, 55.7, 51.9, 51.7, 49.7, 49.6, 42.4, 42.3, 31.3, 31.1, 26.5, 26.4, 25.3, 25.0, 16.3, 16.0

HRMS (ESI) *m/z*: calculated for $C_{26}H_{41}N_8O_{12}S_2^+$ $(M+H)^+$ 721.22799; found: 721.22851

LCMS (ESI) Rt = 1.2 min, observed as $[M+H]^+$

4.4 *cyc*[Ala-βAsp-Cys-Gly]$_2$ (**5**)

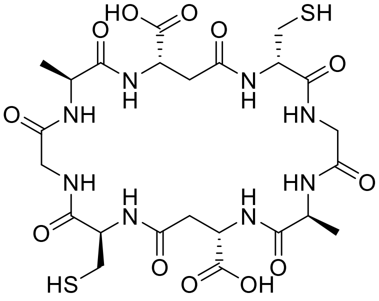

¹H NMR (400 MHz, D_2O) δ (ppm) 4.75 – 4.68 (m, 2H), 4.52 (t, *J* = 6.2 Hz, 2H), 4.35 (q, *J* = 7.0 Hz, 2H), 3.98 (s, 4H), 2.97 – 2.92 (m, 8H), 1.40 (d, *J* = 7.14 Hz, 6H)

¹³C NMR (126 MHz, D_2O) δ (ppm) 174.5, 174.4, 172.7, 172.5, 170.8, 56.0, 49.9, 49.7, 42.4, 37.0, 25.0, 16.5

HRMS (ESI) *m/z*: calculated for $C_{24}H_{37}N_8O_{12}S_2^+$ $(M+H)^+$ 693.19669; found: 693.19735

LCMS (ESI) Rt = 1.1 min, observed as $[M+H]^+$

4.5 *cyc*[Asn-γGlu-Cys-Gly]$_2$ (**8**)

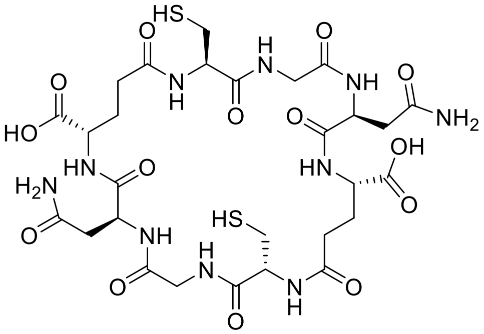

^{1}H NMR (400 MHz, D_2O) δ (ppm) 4.75 - 70 (m, 2H), 4.65 (t, *J* = 6.0 Hz, 1H), 4.60 – 4.54 (m, 1H), 4.37 (dd, *J* = 10.5 Hz, 2H), 4.00 (d*J* = 14.3 Hz, 4H), 2.96 – 2.89 (m, 4H), 2.88 (d, *J* = 5.8 Hz, 1H), 2.84 (d, *J* = 5.6, 1H), 2.78 (d, *J* = 8.1 Hz, 1H), 2.73 (d, *J* = 8.2 Hz, 1H), 2.48 – 2.38 (m, 4H), 2.37 – 2.24 (m, 2H), 2.08 – 1.93 (m, 2H)

^{13}C NMR (126 MHz, D_2O) δ (ppm) 175.1, 174.9, 174.6, 172.5, 172.2, 170.9, 55.3, 51.9, 50.4, 42.5, 35.6, 31.4, 26.4, 25.8

HRMS (ESI) *m/z*: calculated for $C_{28}H_{41}N_{10}O_{14}S_2^-$ (M-H)$^-$ 805.22506; found: 805.22452

LCMS (ESI) Rt = 0.9 min, observed as [M+H]$^+$

4.6 *cyc*[Cya-γGlu-Cys-Gly]$_2$ (**9**)

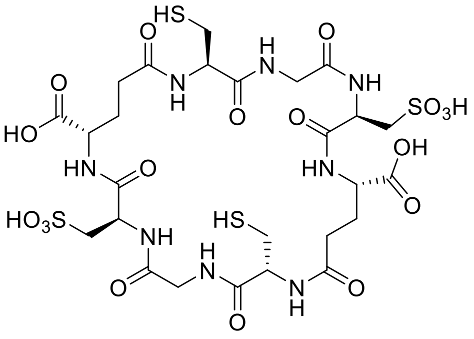

^{1}H NMR (400 MHz, D_2O) δ (ppm) 4.77 – 4.72 (m, 2H), 4.65 (t, *J* = 6.2 Hz, 2H), 4.39 (dd, *J* = 10.6, 3.2 Hz, 2H), 4.00 (d, *J* = 8.5 Hz, 4H), 3.45 – 3.27 (m, 4H), 2.96 – 2.84 (m, 4H), 2.49 – 2.38 (m, 4H), 2.38 – 2.28 (m, 2H), 2.06 – 1.93 (m, 2H)

^{13}C NMR (126 MHz, D_2O) δ (ppm) 179.0, 178.6, 178.4, 176.0, 175.5, 174.1, 174.0, 173.9, 173.8, 58.9, 58.3, 56.3, 56.2, 53.7, 53.5, 53.0, 52.9, 45.8, 45.7, 34.5, 34.4, 29.8, 29.7, 28.6,28.3 (two conformers are observed)

HRMS (ESI) *m/z*: calculated for $C_{26}H_{40}N_8O_{18}NaS_4^+$ $(M+Na)^+$ 903.12356; found: 903.12396

LCMS (ESI) Rt = 0.6 min, observed as $[M+H]^+$

5 *In vitro* studies

5.1 Recovery assay

HT-29 cells were chosen as a representative human cell-line due to their high Pb sensitivity and as their medium, RPMI-1640, is compatible with Pb(II), inhibiting metal precipitation.

HT-29 cells were grown in 25 mM HEPES RPMI-1640 medium, supplemented with 1% L-glutamine, 1% penicillin/streptomycin and 10% fetal calf serum (FCS) superior (standardized) at 37 °C and 5% CO_2. 96-well plates were prepared such that every well contained 10,000 cells in 100 µL medium, and the cells were allowed to adhere for 24 h.

To all wells but the positive control wells, 10 µL of 2.2 mM $Pb(NO_3)_2$ were added (final concentration 0.2 mM). 10 µL of H_2O were added to the positive control wells. 60 min after the addition of the metal salt, 10 µL of each solution of the examined peptide (0.24, 0.6, 1.2, 2.4, 4.8 and 12 mM) were added to reach final concentrations of 0.02, 0.05, 0.1, 0.2, 0.4 and 1.0 mM (which are 0.1, 0.25, 0.5, 1, 2 and 5 equivalents, respectively). To the positive control wells containing no metal and the negative control wells containing metal but no peptides, 10 µL of H_2O were added, respectively. Each condition was performed in triplicates.

The plates were incubated at 37 °C, and 5% CO_2 for additional 23 h, after which the medium was removed, the wells were washed with H_2O, and 50 µL of crystal violet solution[10,11] (500 mg crystal violet powder in 20 mL CH_3OH and 80 mL H_2O) were added to each well. The plates were gently shaken (50 rpm) for 20 min. The plates were then washed with H_2O until no more unbound dye was observed and allowed to dry overnight. 100 µL of CH_3OH were added to each well, and the plates were gently shaken (50 rpm) for 20 min, after which their absorbance at 560 nm was recorded.

The recovery values of each concentration of a peptide were calculated according to Equation S4:

$$Recovery\% = \frac{A[Pep_X]-A[blank]}{A[NEG]-A[blank]} \times 100\% \quad \text{(S4)}$$

$A[Pep_X]$ – absorbance of the pre-toxified well in the presence of X mM of peptide

A[blank] – absorbance of an empty well

A[NEG] – absorbance of the pre-toxified well that was not treated with any peptide

Each experiment was performed on three independent repeats. Values are mean ± SD.

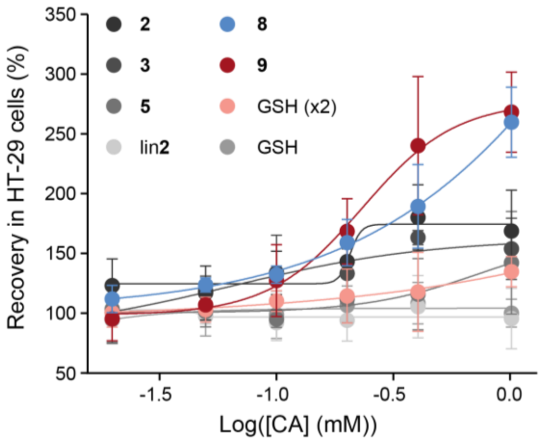

Figure S2. Dose-dependent recovery of HT-29 cells treated with $Pb(NO_3)_2$ (0.2 mM) followed by the administration of the five peptides, GSH, and GSH at double the concentration (GSH (x2); 1 h after the addition of Pb(II) ions; values are calculated relative to Pb-poisoned cells as the negative control for the recovery). Values are mean ± SD of at least 3 repeats.

5.2 Toxicity assay

96-well plates were prepared such that every well contained 10,000 HT-29 cells in 100 µL medium, and the cells were allowed to adhere for 24 h.

To all wells but the positive control wells, 10 µL of each solution of the examined peptides (0.24, 0.6, 1.2, 2.4, 4.8 and 12 mM) were added to reach final concentrations of 0.02, 0.05, 0.1, 0.2, 0.4 and 1.0 mM. To the positive control wells, 10 µL of H_2O were added. Each condition was

performed in triplicates. The plates were incubated at 37 °C, and 5% CO_2 for 23 h, after which the medium was removed, the wells were washed with H_2O, and 50 µL of crystal violet solution[10] (500 mg crystal violet powder in 20 mL CH_3OH and 80 mL H_2O) were added to each well. The plates were gently shaken (50 rpm) for 20 min. The plates were then washed with H_2O until no more unbound dye was observed and allowed to dry overnight. 100 µL of CH_3OH were added to each well, and the plates were gently shaken (50 rpm) for 20 min, after which their absorbance at 560 nm was recorded.

The toxicity of each concentration of a peptide was calculated according to Equation S5:

$$Toxicity\% = \frac{A[Pep_X]-A[blank]}{A[POS]-A[blank]} \times 100\% \tag{S5}$$

$A[Pep_X]$ – absorbance of well in the presence of X mM of peptide

A[blank] – absorbance of an empty well

A[POS] – absorbance of well that contains no peptides

Each experiment was performed on three independent repeats. Values are mean ± SD.

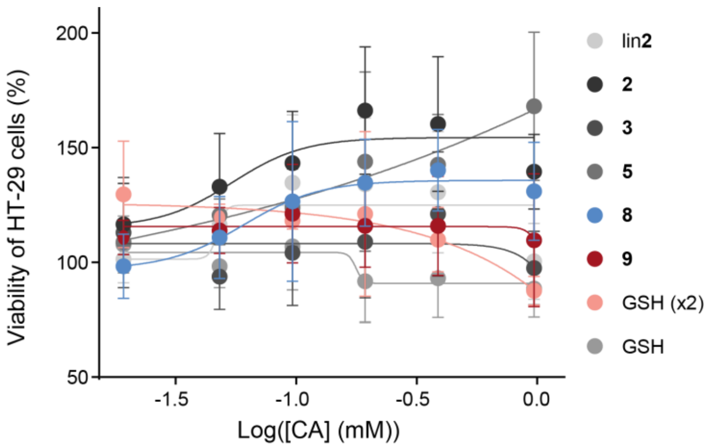

Figure S3. Dose-dependent viability of HT-29 cells treated with the five peptides, GSH, and GSH at double the concentration (GSH (x2)). Values are mean ± SD of at least 3 repeats.

5.3 DCF assay

ROS production determination in the presence of Pb(II) ions

96-well plates were prepared such that every well contained 5,000 cells in 50 µL medium, and the cells were allowed to adhere overnight.

To all wells but the positive control wells, 5 µL of 2.2 mM $Pb(NO_3)_2$ were added (final concentration 0.2 mM). 5 µL of H_2O were added to the positive control wells. 1 h after, 5 µL of **8** or GSH (12 mM) were added to reach final concentrations of 1 mM (5 equiv.). To the positive control wells containing no metal and the negative control wells containing metal but no peptide, 5 µL of H_2O were added.

The plates were incubated at 37 °C, and 5% CO_2 for additional 23 h, after which the medium was removed, the wells were washed with PBS, and 50 µL of PBS were added to each well. 6.75 µL of 2',7'-dichlorodihydrofluorescein diacetate (H_2DCF-DA) (2.1 mM in DMSO) were added (final concentration 0.25 mM) and gently mixed. The plates were incubated for an additional 1 h. Fluorescence was then measured at an excitation of 490 nm and an emission of 520 nm.

ROS production determination in the absence of Pb(II) ions

96-well plates were prepared such that every well contained 5,000 cells in 50 µL medium, and the cells were allowed to adhere overnight.

To all wells but the positive control wells, 5 µL of **8** or GSH (12 mM) were added to reach final concentrations of 1 mM. To the positive control wells, 5 µL of H_2O were added.

The plates were incubated at 37 °C, and 5% CO_2 for additional 23 h, after which the medium was removed, the wells were washed with PBS, and 50 µL of PBS were added to each well. 6.75 µL of 2',7'-dichlorodihydrofluorescein diacetate (H_2DCF-DA) (2.1 mM in DMSO) were added (final concentration 0.25 mM) and gently mixed. The plates were incubated for an additional 1 h. Fluorescence was then measured at an excitation of 490 nm and an emission of 520 nm.

6 Complexes characterization

6.1 Determination of complexation stoichiometries of **8** with $Pb(NO_3)_2$ with HR-ESI-MS

Table S4 Detected HR-ESI-MS signals in the equimolar mixture of **8** and $Pb(NO_3)_2$ (100 µM)

**Peak**	***m/z***	**Adduct**	**Deprotonation of ligand**
A	529	[**8**+Pb+2Na]$^{2+}$	2
B	610	[**8**+2Pb+2H]$^{2+}$	4
C	712	[**8**+3Pb]$^{2+}$	4
D	829	[**8**+Na]$^{+}$	0
E	1035	[**8**+Pb+Na]$^{+}$	2
F	1218	[**8**+2Pb+H]$^{+}$	4

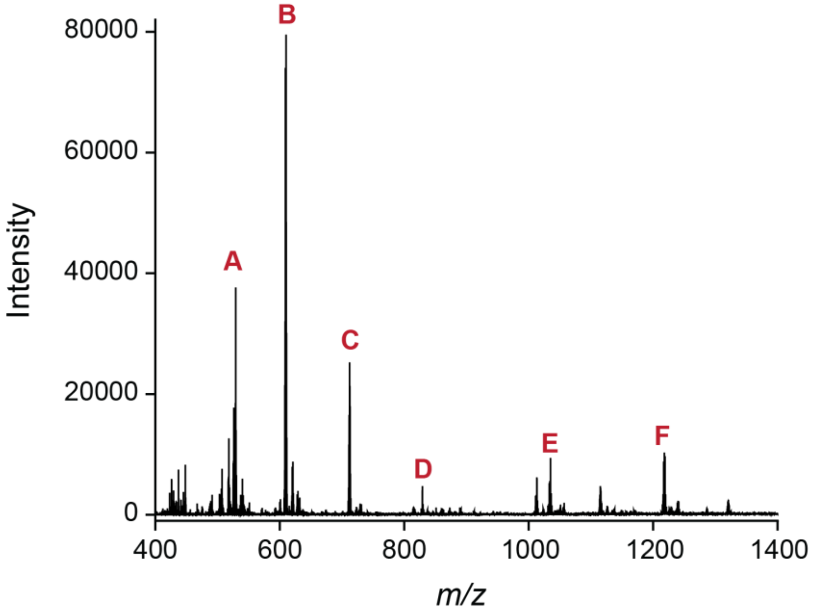

Figure S4. Full positive HR-ESI-MS spectra (*m/z* 400-1400) of **8** (100 µM) incubated with $Pb(NO_3)_2$ (100 µM) in H_2O.

6.2 Determination of complex speciation of **8** with $ZnCl_2$ by HR-ESI-MS

Table S5 Detected HR-ESI-MS signals in the equimolar mixture of **8** and $ZnCl_2$ (100 µM)

Peak	*m/z*	Adduct	Deprotonation of ligand
A	423	[**8**+K+H]$^{2+}$	0
B	442	[**8**+2K]$^{2+}$	0
C	454	[**8**+K+Zn]$^{2+}$	1
D	467	[**8**+2Zn]$^{2+}$	2
E	807	[**8**+H]$^{+}$	0
F	829	[**8**+Na]$^{+}$	0
G	845	[**8**+K]$^{+}$	0
H	869	[**8**+Zn]$^{+}$	1

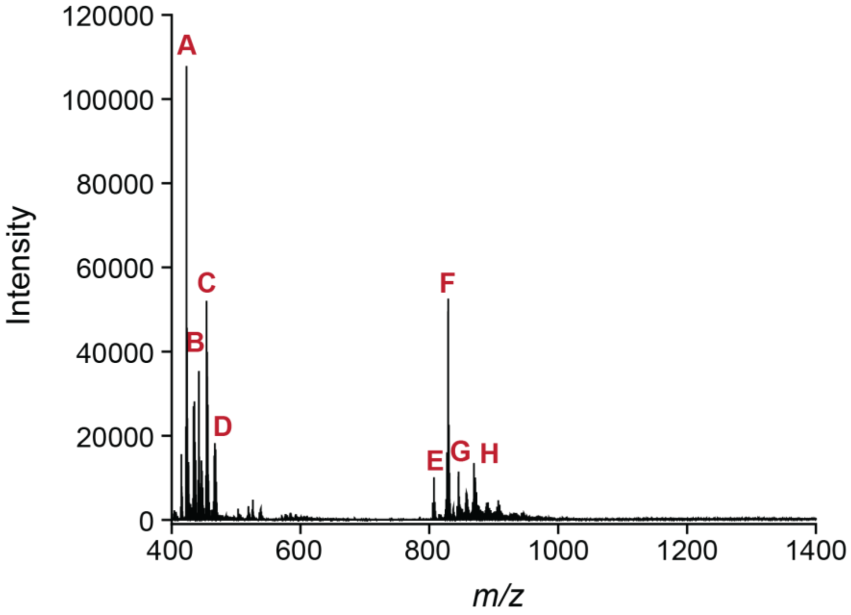

Figure S5. Full positive HR-ESI-MS spectra (*m/z* 400-1400) of **8** (100 µM) incubated with $ZnCl_2$ (100 µM) in H_2O.

6.3 Determination of complex speciation of **8** with $Pb(NO_3)_2$ and $ZnCl_2$ by HR-ESI-MS

Table S6 Detected HR-ESI-MS signals in the equimolar mixture of **8**, $Pb(NO_3)_2$ and $ZnCl_2$ (100 µM)

**Peak**	***m/z***	**Adduct**	**Deprotonation of ligand**
A	435	[**8**+Zn+2H]$^{2+}$	2
B	467	[**8**+2Zn+2H]$^{2+}$	4
C	538	[**8**+Pb+Zn+2H]$^{2+}$	4
D	610	[**8**+2Pb+2H]$^{2+}$	4
E	641	[**8**+2Pb+Zn+2H]$^{2+}$	6
F	712	[**8**+3Pb+2H]$^{2+}$	6
G	869	[**8**+Zn+H]$^{+}$	2
H	1075	[**8**+Pb+Zn+H]$^{+}$	4
I	1218	[**8**+2Pb+H]$^{+}$	4

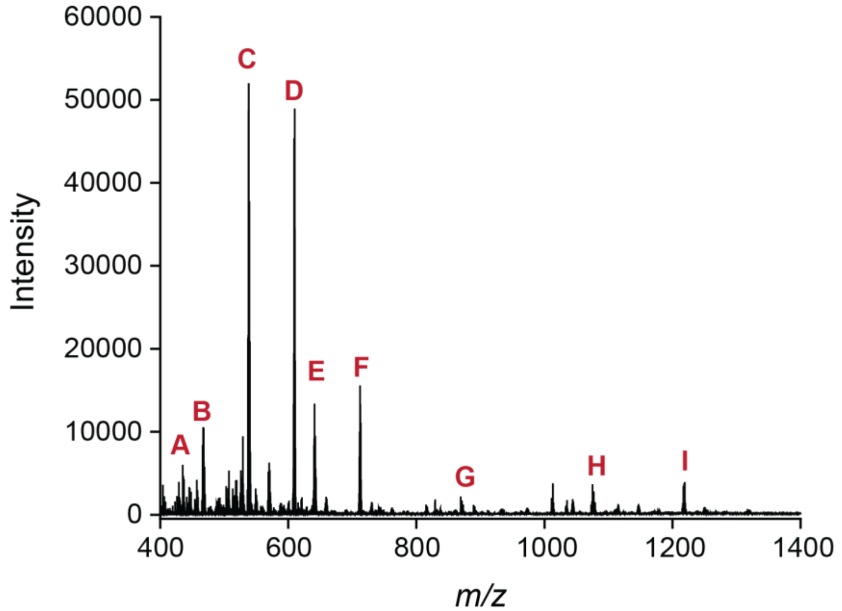

Figure S6. Full positive HR-ESI-MS spectra (*m/z* 400-1400) of **8** (100 µM) incubated with $Pb(NO_3)_2$ (100 µM) and $ZnCl_2$ (100 µM) in H_2O.

Table S7 Detected HR-ESI-MS signals in the equimolar mixture of **8**, $Pb(NO_3)_2$ and $ZnCl_2$ (300 μM)

**Peak**	***m/z***	**Adduct**	**Deprotonation of ligand**
A	423	[**8**+K+H]$^{2+}$	0
B	435	[**8**+Zn+2H]$^{2+}$	2
C	454	[**8**+K+Zn]$^{2+}$	1
D	467	[**8**+2Zn]$^{2+}$	2
E	538	[**8**+Pb+Zn+2H]$^{2+}$	4
F	610	[**8**+2Pb+2H]$^{2+}$	4
G	641	[**8**+2Pb+Zn]$^{2+}$	4
H	712	[**8**+Pb+Zn+H]$^{+}$	4
I	807	[**8**+H]$^{+}$	0
J	869	[**8**+Zn+H]$^{+}$	2
K	1013	[**8**+Pb+H]$^{+}$	2
L	1075	[**8**+Pb+Zn+H]$^{+}$	4

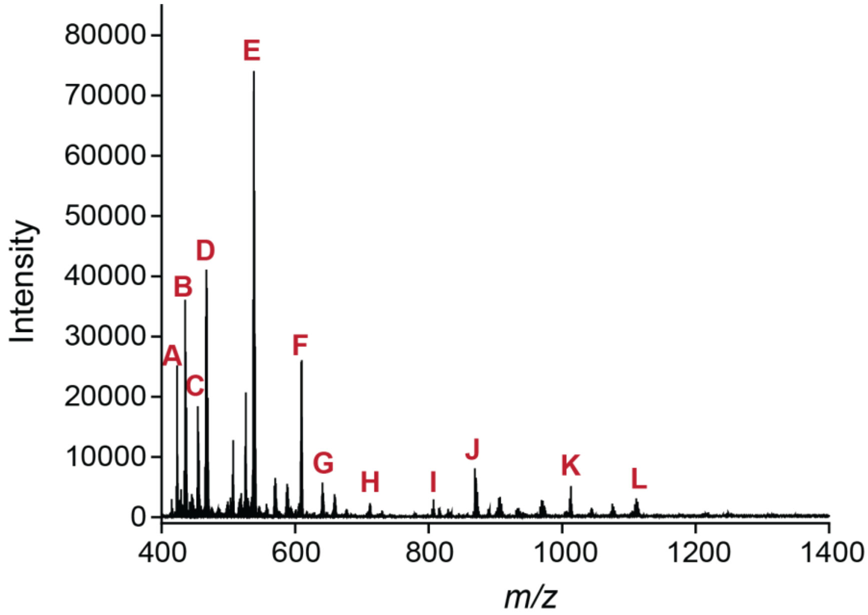

Figure S7. Full positive HR-ESI-MS spectra (*m/z* 400-1400) of **8** (100 µM) incubated with $Pb(NO_3)_2$ (100 µM) and $ZnCl_2$ (300 µM) in H_2O.

Table S8 Detected HR-ESI-MS signals in the equimolar mixture of **8**, $Pb(NO_3)_2$ and $ZnCl_2$ (500 µM)

Peak	*m/z*	Adduct	Deprotonation of ligand
A	423	[**8**+K+H]$^{2+}$	0
B	435	[**8**+Zn+2H]$^{2+}$	2
C	454	[**8**+K+Zn]$^{2+}$	1
D	467	[**8**+2Zn]$^{2+}$	2
E	538	[**8**+Pb+Zn+2H]$^{2+}$	4
F	610	[**8**+2Pb+2H]$^{2+}$	4
G	641	[**8**+2Pb+Zn]$^{2+}$	4
H	807	[**8**+H]$^{+}$	0
I	869	[**8**+Zn+H]$^{+}$	2
J	1013	[**8**+Pb+H]$^{+}$	2
K	1075	[**8**+Pb+Zn+H]$^{+}$	4

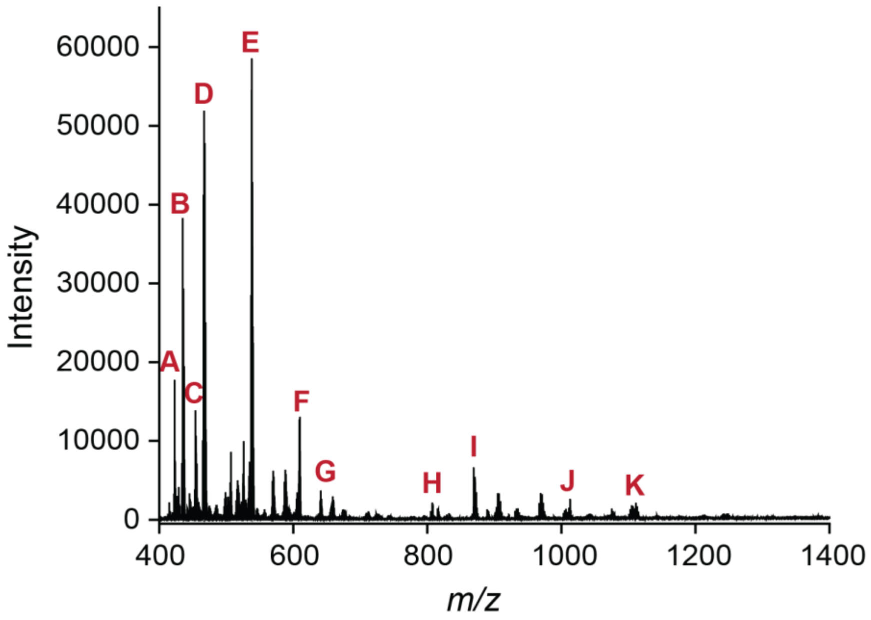

Figure S8. Full positive HR-ESI-MS spectra (*m/z* 400-1400) of **8** (100 µM) incubated with $Pb(NO_3)_2$ (100 µM) and $ZnCl_2$ (500 µM) in H_2O.

6.4 Affinity determination with $Pb(NO_3)_2$ by ITC

Titrations were performed in 20 mM Tris buffer pH 6.5 at 25 °C, where $Pb(NO_3)_2$ solution (280 µL) was titrated to a peptide solution (1.8 mL) at concentrations according to Table S9.

Table S9 Peptides and $Pb(NO_3)_2$ concentrations for ITC-monitored titrations

	**[Peptide] (µM)**	**[$Pb(NO_3)_2$] (mM)**
**8**	50	12.5
**9**	200	4.0

6.5 UV-Vis titration of $Pb(NO_3)_2$ with peptides

To aliquots of **8** or **9** (100 µM) in 20 mM Tris buffer pH 6.5, $Pb(NO_3)_2$ was added at final concentrations of 25 –400 µM (equal to 0.25 – 4 equivalents), and the total volume was brought to 200 µL with Tris buffer. The spectrum of each solution was recorded (200-400 nm), and a blank spectrum of only buffer was omitted from all spectra.

6.6 Determination of Pb-complex stability by UV-Vis titrations

To aliquots of **8** or **9** and $Pb(NO_3)_2$ at final concentrations of 100 µM in 20 mM Tris buffer pH 6.5 $ZnCl_2$ (2 mM) or $CaCl_2$ (20 mM) were added after 1 h in 1-10 equivalents for $ZnCl_2$ or 10-100 equivalents for $CaCl_2$, and the total volume was brought to 200 µL with Tris buffer. The spectrum of each solution was recorded (200-400 nm), and a blank spectrum of only buffer was omitted from all spectra.

6.7 Determination of metal selectivity by UV-Vis titrations

To aliquots of **8** or **9** and $ZnCl_2$ (1 mM) or $CaCl_2$ (10 mM) in 20 mM Tris buffer pH 6.5 $Pb(NO_3)_2$ was added after 1 h in 1-1 equivalents compared with the peptide's concentration (100 µM), and the total volume was brought to 200 µL with Tris buffer. The spectra of each solution were recorded (200-400 nm), and a blank spectrum of only buffer was omitted from all spectra.

7 Ellman’s test

Calibration curve: to a 96-well plate with freshly-prepared solutions of Ac-Cys-OMe (12.5-200 µM) in H_2O supplemented with 10% 1 M Tris buffer pH 8.0, a fresh solution of Ellman’s reagent (5,5'-dithiobis-(2-nitrobenzoic acid; DTNB; final concentration of 300 µM in H_2O supplemented with 12.5 mM sodium acetate) was added.[12] The absorbance of each well at 412 nm was then determined. The average and standard deviation within six wells containing the same AA concentration were calculated and linearly plotted using Origin software.

Seven 96-well plates were prepared identically with fresh solutions of **8** (100 µM) in H_2O supplemented with 10% 1 M Tris buffer pH 8.0, where each condition is replicated in 3 wells. A fresh solution of Ellman’s reagent (DTNB; final concentration of 300 µM in H_2O supplemented with 12.5 mM sodium acetate) was added at different intervals: t = 0, 1, 2, 4, 6, 12, 24 h.[12] The absorbance of each well at 412 nm was then determined, and the concentration of free thiol per well was calculated according to the calibration curve. For each time frame, the average and standard deviation were calculated. The percentage of the reduced peptide was calculated according to Equation S6:

$$Reduced\ peptide\% = \frac{[peptide_{t=x}]}{[peptide_{t=0}]} \times 100\% \qquad \text{(S6)}$$

$[peptide_{t=x}]$ – concentration of the peptide solution at t = x (x > 0)

$[peptide_{t=0}]$ – concentration of the peptide solution at t = 0

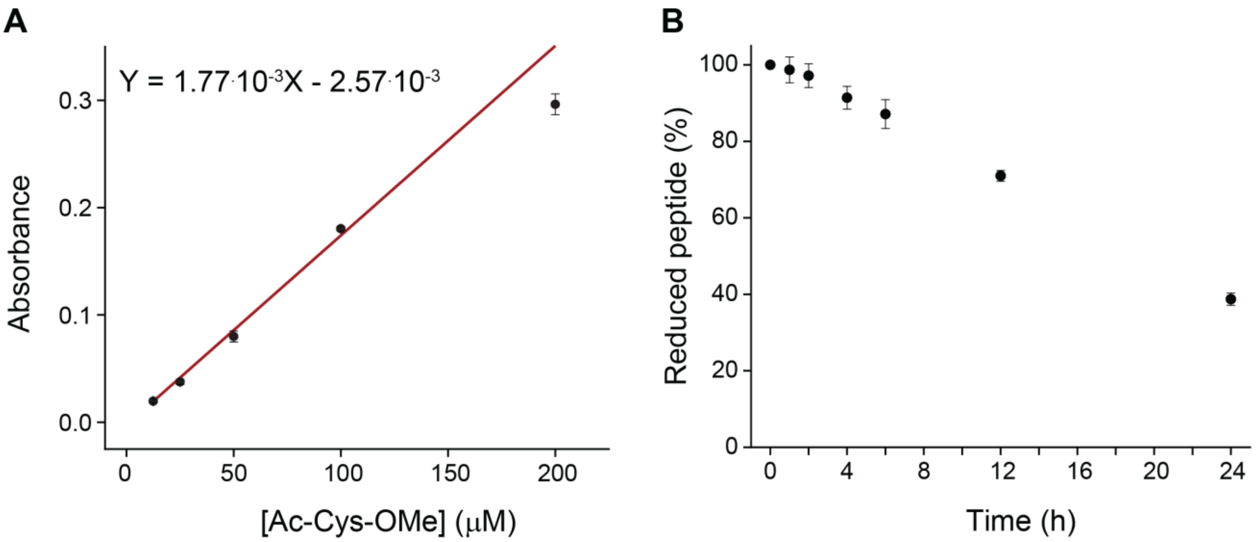

Figure S9. Calibration curve of Ac-Cys-OMe at various concentrations in H_2O reacted with Ellman's reagent (at excess) and measured immediately (A); **8** (100 μM) at H_2O incubated at RT for various times frames and reacted with Ellman's reagent (300 μM; B).

8 Mode of action

96-well plates were prepared such that every well contained 10,000 HT-29 cells in 100 µL medium, and the cells were allowed to adhere for 24 h. Cells with Pb(II) ions only were analyzed after 1 or 24 h incubation with the metal salt as reference values:

1 h Incubation

To all wells, 10 µL of 2.2 mM $Pb(NO_3)_2$ were added (final concentration 0.2 mM) and the cells were incubated at 37 °C, and 5% CO_2 for 1 h, after which samples were prepared for analysis by ICP-MS.

24 h Incubation

To all wells, 10 µL of 2.2 mM $Pb(NO_3)_2$ were added (final concentration 0.2 mM) followed by incubation at 37 °C, and 5% CO_2 for 1 h. 10 µL of H_2O were then added to the wells, followed by further incubation at 37 °C, and 5% CO_2 for additional 23 h, after which samples were prepared for analysis by ICP-MS.

Cells treated with **8** were analyzed in two sets that differed in the order of peptide and metal salt addition:

8 added after poisoning with Pb

To all wells, 10 µL of 2.2 mM $Pb(NO_3)_2$ were added (final concentration 0.2 mM) and the cells were incubated at 37 °C, and 5% CO_2 for 1 h. 10 µL solution of **8** in H_2O (12 mM) were added to reach a final concentration of 1.0 mM (5 equivalents). The cells were incubated at 37 °C, and 5% CO_2 for additional 23 h, after which samples were prepared for analysis by ICP-MS.

8 added before poisoning with Pb

To all wells, 10 µL solution of **8** in H_2O (12 mM) were added to reach a final concentration of 1.0 mM and the cells were incubated at 37 °C, and 5% CO_2 for 1 h. 10 µL of 2.2 mM $Pb(NO_3)_2$ were added (final concentration 0.2 mM) and the cells were incubated at 37 °C, and 5% CO_2 for additional 23 h, after which samples were prepared for analysis by ICP-MS.

Sample preparation for ICP-MS analysis

After respective incubation times, the supernatants were homogenized by gently pipetting up and down and 50 µL were transferred and digested with 100 µL HNO_3 (70%) for 1 h in a 15 mL tube. 42 µL of Lu_2O_3 (25 mg L^{-1} in 2% HNO_3) were added as internal standard, followed by the addition of 3.808 mL H_2O. Pb in the supernatant was then quantified using ICP-MS.

Since the cells are adherent, residual supernatant was removed from each well and the cells were washed with RPMI (3x200 µL). To each well, 100 µL of HNO_3 (70%) were added and the cells were digested for 1 h at rt. 50 µL of the resulting solution were taken and further 100 µL of HNO_3 (70%) were added. 42 µL of Lu_2O_3 (25 mg L^{-1} in 2% HNO_3) were added as internal standard, followed by the addition of 3.808 mL H_2O. Pb in the cells was then quantified using ICP-MS.

For each condition three independent experiments were conducted.

Table S10 Ratio of Pb content in ppb found in supernatants (SN) and cells (C)

**Condition**	**1**	**2**	**3**	**4**
**SN/C**	99.9 ± 65.4	119.0 ± 21.0	56.3 ± 27.5	43.3 ± 10.23

(1) Cells poisoned with 0.2 mM $Pb(NO_3)_2$ for 1 h, (2) cells poisoned with 0.2 mM $Pb(NO_3)_2$ and incubated for 24 h, (3) cells were poisoned with 0.2 mM $Pb(NO_3)_2$ and incubated for 1 h, followed by the addition of 1 mM **8** and incubation for additional 23 h, (4) cells were given 1 mM **8** and incubated for 1 h, followed by the addition of 0.2 mM $Pb(NO_3)_2$ and incubation for additional 23 h.

9 Stability in human blood serum

A freshly prepared solution of **8** (in H_2O) was added to the human blood serum (HBS, purchased from Sigma Aldrich) to reach a final peptide concentration of 100 µM and a volume of 1 mL. Samples were pipetted carefully up and down three times and incubated at 37 °C. Aliquots (50 µL) were taken periodically, starting from the addition of the serum (0 min) and continuing after 1 h up to 48 h. Aliquots were poured into a mixture of CH_3CN:H_2O (1:1; 200 µL) to precipitate the HBS proteins. The samples were cooled on ice for 30 min and centrifuged at 4’400 rpm for 10 min. The supernatant was analyzed by LCMS.

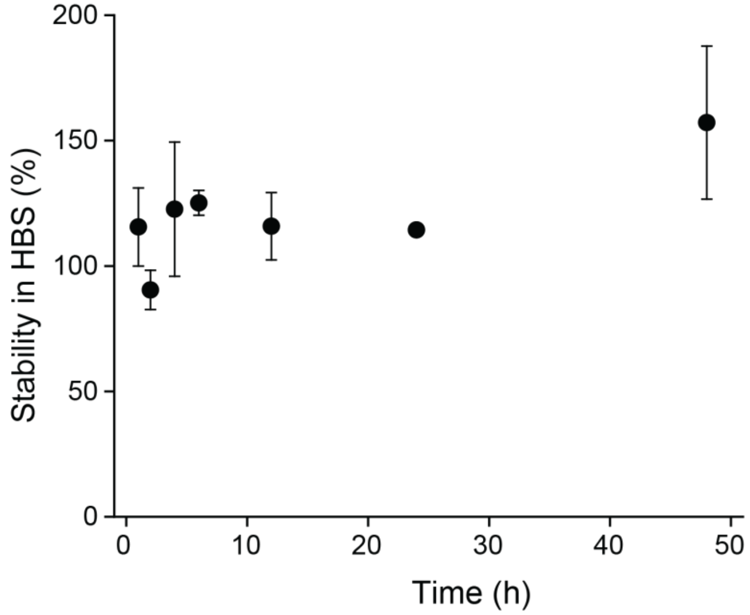

Figure S10. Stability of **8** in human blood serum.

10 References

[1] S. G. Balasubramani, G. P. Chen, S. Coriani, M. Diedenhofen, M. S. Frank, Y. J. Franzke, F. Furche, R. Grotjahn, M. E. Harding, C. Hättig, A. Hellweg, B. Helmich-Paris, C. Holzer, U. Huniar, M. Kaupp, A. Marefat Khah, S. Karbalaei Khani, T. Müller, F. Mack, B. D. Nguyen, S. M. Parker, E. Perlt, D. Rappoport, K. Reiter, S. Roy, M. Rückert, G. Schmitz, M. Sierka, E. Tapavicza, D. P. Tew, C. Van Wüllen, V. K. Voora, F. Weigend, A. Wodyński, J. M. Yu, *J. Chem. Phys.* **2020**, *152*.

[2] A. D. Becke, *Phys. Rev. A* **1988**, *38*, 3098.

[3] S. H. Vosko, L. Wilk, M. Nusair, *Can. J. Phys.* **1980**, *58*, 1200–1211.

[4] J. P. Perdew, *Phys. Rev. A* **1986**, *33*, 8822–8824.

[5] S. Grimme, *Chem. - A Eur. J.* **2012**, *18*, 9955–9964.

[6] F. Weigend, R. Ahlrichs, *Phys. Chem. Chem. Phys.* **2005**, *7*, 3297–3305.

[7] A. Klamt, G. Schüürmann, *J. Chem. Soc. Perkin Trans. 2* **1993**, *5*, 799–805.

[8] T. A. Mohammed, C. M. Meier, T. Kalvoda, M. Kalt, L. Rulíšek, M. S. Shoshan, *Angew. Chem. Int. Ed.* **2021**, *60*, 12381–12385.

[9] A. Klamt, *J. Phys. Chem.* **1995**, *99*, 2224–2235.

[10] M. Feoktistova, P. Geserick, M. Leverkus, *Cold Spring Harb. Protoc.* **2016**, *2016*, 343–346.

[11] Viability assays that are based on reduction, such as MTT, are not suited due to the ability of thiols to reduce the probes.

[12] C. K. Riener, G. Kada, H. J. Gruber, *Anal. Bioanal. Chem.* **2002**, *373*, 266–276.
